# *Onchocerca volvulus* microfilariae in the anterior chambers of the eye and ocular adverse events after a single dose of 8 mg moxidectin or 150 µg/kg ivermectin: results of a randomized double-blind Phase 3 trial in the Democratic Republic of the Congo, Ghana and Liberia

**DOI:** 10.1186/s13071-023-06087-3

**Published:** 2024-03-15

**Authors:** Eric M. Kanza, Amos Nyathirombo, Jemmah P. Larbelee, Nicholas O. Opoku, Didier K. Bakajika, Hayford M. Howard, Germain L. Mambandu, Maurice M. Nigo, Deogratias Ucima Wonyarossi, Françoise Ngave, Kambale Kasonia Kennedy, Kambale Kataliko, Kpehe M. Bolay, Simon K. Attah, George Olipoh, Sampson Asare, Mupenzi Mumbere, Michel Vaillant, Christine M. Halleux, Annette C. Kuesel

**Affiliations:** 1 Centre de Recherche Clinique de Butembo, Université Catholique du Graben, Site Horizon, Butembo, Nord Kivu Democratic Republic of the Congo; 2 Centre de Recherche en Maladies Tropicale de L’Ituri, Hôpital Générale de Référence de Rethy, Ituri, Democratic Republic of the Congo; 3 Clinical Research Center, Liberia Institute for Biomedical Research, Bolahun, Liberia; 4 Onchocerciasis Chemotherapy Research Center, Hohoe, Ghana; 5 Competence Center for Methodology and Statistics, Luxembourg Institute of Health, Strassen, Grand Duchy of Luxembourg; 6 UNICEF/UNDP/World Bank/WHO Special Programme for Research and Training in Tropical Diseases (WHO/TDR), World Health Organization, Geneva, Switzerland; 7 Present Address: Programme National de Lutte Contre Les Maladies Tropicales Négligées À Chimio-Thérapie Préventive (PNLMTN-CTP), Kinshasa, Democratic Republic of the Congo; 8 Present Address: Department of Ophthalmology, Faculty of Medicine, Gulu University, Gulu, Uganda; 9 Present Address: Ministry of Health, Monrovia, Liberia; 10 Present Address: Department of Epidemiology and Biostatistics School of Public Health, University of Health and Allied Sciences, Hohoe, Ghana; 11 Present Address: ESPEN, African Regional Office of the World Health Organization (WHO/AFRO/ESPEN), Brazzaville, Republic of Congo; 12 Present Address: Ganta United Methodist Hospital, Ganta City, Nimba County Liberia; 13 Present Address: Inspection Provinciale de La Santé de La Tshopo, Division Provinciale de La Santé de La Tshopo, Kisangani, Province de La Tshopo Democratic Republic of the Congo; 14 Present Address: Institut Supérieur Des Techniques Médicales de Nyankunde, Bunia, Ituri Democratic Republic of the Congo; 15 Present Address: Department of Clinical Research, London School of Hygiene and Tropical Medicine, London, UK; 16 Present Address: Centre de Santé CECA 20 de Mabakanga, Beni, Nord Kivu Democratic Republic of the Congo; 17 Present Address: National Public Health Institute of Liberia, Public Health & Medical Research, Monrovia, Liberia; 18 Present Address: Department of Microbiology, University of Ghana Medical School, Accra, Ghana; 19 Present Address: Baldwin University College, Accra, Ghana; 20 Present Address: National Assay Centre, Precious Minerals Marketing Company Ltd., Diamond House, Accra, Ghana; 21 Present Address: Bell Laboratories Inc, Window, WI USA; 22 Present Address: Medicines Development for Global Health (MDGH), Melbourne, Australia

**Keywords:** Onchocerciasis, Moxidectin, Ivermectin, Diethylcarbamazine, Ocular microfilariae, Microfilariae in the anterior chamber, Increase in ocular microfilariae, Microfilariae mobilization, Ocular Mazzotti reactions, Ocular adverse events

## Abstract

**Background:**

After ivermectin became available, diethylcarbamazine (DEC) use was discontinued because of severe adverse reactions, including ocular reactions, in individuals with high *Onchocerca volvulus* microfilaridermia (microfilariae/mg skin, SmfD). Assuming long-term ivermectin use led to < 5 SmfD with little or no eye involvement, DEC + ivermectin + albendazole treatment a few months after ivermectin was proposed. In 2018, the US FDA approved moxidectin for treatment of *O. volvulus* infection. The Phase 3 study evaluated SmfD, microfilariae in the anterior chamber (mfAC) and adverse events (AEs) in ivermectin-naïve individuals with ≥ 10 SmfD after 8 mg moxidectin (*n* = 978) or 150 µg/kg ivermectin (*n* = 494) treatment.

**Methods:**

We analyzed the data from 1463 participants with both eyes evaluated using six (0, 1–5, 6–10, 11–20, 21–40, > 40) mfAC and three pre-treatment (< 20, 20 to < 50, ≥ 50) and post-treatment (0, > 0–5, > 5) SmfD categories. A linear mixed model evaluated factors and covariates impacting mfAC levels. Ocular AEs were summarized by type and start post-treatment. Logistic models evaluated factors and covariates impacting the risk for ocular AEs.

**Results:**

Moxidectin and ivermectin had the same effect on mfAC levels. These increased from pre-treatment to Day 4 and Month 1 in 20% and 16% of participants, respectively. Six and 12 months post-treatment, mfAC were detected in ≈5% and ≈3% of participants, respectively. Ocular Mazzotti reactions occurred in 12.4% of moxidectin- and 10.2% of ivermectin-treated participants without difference in type or severity. The risk for ≥ 1 ocular Mazzotti reaction increased for women (OR 1.537, 95% CI 1.096–2.157) and with mfAC levels pre- and 4 days post-treatment (OR 0: > 10 mfAC 2.704, 95% CI 1.27–5.749 and 1.619, 95% CI 0.80–3.280, respectively).

**Conclusions:**

The impact of SmfD and mfAC levels before and early after treatment on ocular AEs needs to be better understood before making decisions on the risk-benefit of strategies including DEC. Such decisions should take into account interindividual variability in SmfD, mfAC levels and treatment response and risks to even a small percentage of individuals.

**Graphical Abstract:**

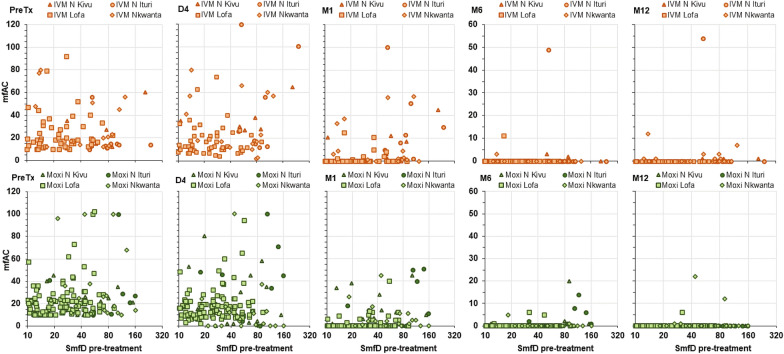

**Supplementary Information:**

The online version contains supplementary material available at 10.1186/s13071-023-06087-3.

## Background

Onchocerciasis is a vector-borne parasitic disease caused by the parasite *Onchocerca volvulus*, transmitted by *Simulium* black flies. The morbidity, due primarily to immunological reactions of the body to the dead microfilariae in the skin and the eyes, and resulting socio-economic effects, have motivated large-scale control and elimination programmes in sub-Saharan Africa, Yemen and the Americas [1–7]. Today, control and elimination programmes are based on mass drug administration of ivermectin (MDAi).

In 2003, an international consultation concluded that parasite elimination through MDAi was feasible in the six Central and South American countries of the Americas (total population at risk around 0.56 million), but not across the vast and partly hyperendemic areas in sub-Saharan Africa [8], requiring research for new drugs or drug combinations [9, 10]. Since then, onchocerciasis has been eliminated in the Americas in all but the large endemic area crossing the Venezuela-Brazil border. This was achieved through between 23 and 36 rounds of biannual MDAi complemented with quarterly MDAi in around 300 communities [7, 11–15]. Data obtained in Africa since 2003 have shown that long-term MDAi, implemented as community-directed treatment (CDTI), may have eliminated transmission or may be close to doing so in many areas [14–18]. These data motivated the objective to eliminate *O. volvulus* transmission in some African countries by 2020 and in 80% of endemic countries by 2025 [19]. These targets have recently been revised to achieve WHO-verified interruption of parasite transmission in 12 countries worldwide and stop MDAi in at least one focus in 34 countries by 2030 [20].

Expert consultations and data reviews by the African Programme for Onchocerciasis Control (APOC, 1995–2015) concluded that alternative treatment strategies (ATS) are required for onchocerciasis elimination in many areas in Africa [19]. Potential ATS identified include those based on moxidectin which was approved in 2018 for treatment of *O. volvulus* infected individuals ≥ 12 years of age by the US Food and Drug Administration (FDA) [21–30]. Discovery, development and implementation research for other ATS [31] is also ongoing including for new or repurposed drugs [10, 32–36], for effective, affordable and sustainable complementary vector control strategies [37, 38] and for approaches to safe use of ivermectin in loiasis co-endemic areas (drugs for safe reduction of *Loa loa* microfilaraemia [39–41] and ‘test-and-not-treat’ or ‘test-and-treat’ strategies [42–46]). Furthermore, research for a vaccine is continuing [47].

The triple drug combination ivermectin, albendazole and diethylcarbamazine (IDA) was recommended in 2017 by WHO as an alternative MDA regimen for elimination of lymphatic filariasis in specific areas [48]. Treatment with IDA following treatment with ivermectin (referred to as ‘pretreat and treat’ strategy) has been proposed as an ATS for onchocerciasis-endemic areas with long-term CDTI to accelerate onchocerciasis elimination if a single IDA dose sterilizes adult *O. volvulus* worms and eliminates microfilariae from the skin permanently [49].

Oral diethylcarbamazine (DEC) was used for onchocerciasis treatment before ivermectin became available. Treatment schedules differed with total doses ranging from 100 to 6000 mg and the number of administrations ranging from a single dose to daily doses for up to 2 weeks. WHO recommended a 0.5 mg/kg or 1.0 mg/kg starting dose for 1–2 days and a 2.0 mg/kg maintenance dose twice a day for 5–7 days for a total dose of approximately 30 mg/kg for adults [50]. While DEC has low intrinsic toxicity [51, 52], oral DEC treatment of *O. volvulus*-infected individuals can result in very severe Mazzotti reactions, the signs and symptoms of the immunological reaction of the body to dead and dying microfilariae. These include worsening of onchocercal eye disease and new ocular lesions and anaphylactic reactions, which resulted in the suggestion to start DEC treatment in the hospital since the susceptibility of an individual is not predictable [50, 53–58]. The severity of the Mazzotti reactions increases with the microfilariae levels in individuals treated with the same oral DEC dose and increases with the DEC dose when individuals with similar microfilariae burden are compared. These observations led to the conclusion that the severity of the Mazzotti reaction after DEC treatment is a function of the number of microfilariae present before treatment and killed [51, 59]. In 1995, the WHO Expert Committee on Onchocerciasis Control recommended that DEC should no longer be used for treatment of onchocerciasis and, if used for treatment of lymphatic filariasis in patients with onchocerciasis, ivermectin should be administered first and DEC only given after skin and ocular mf have been cleared [58]. The proposed ‘pretreat and treat’ strategy to use IDA in onchocerciasis-endemic areas after long-term MDA with ivermectin plus albendazole or long-term CDTI [49] is based on the assumption that long-term MDAi will have reduced *O. volvulus* skin microfilariae density (mf/mg skin, SmfD) to ‘usually less than 5 mf/mg skin’. This would reduce the risk of severe Mazzotti reactions, and this risk would be further reduced by treating only those individuals with IDA who took the ivermectin pretreatment [49]. Given data on the percentage of the population that participates in MDAi and long-standing challenge to optimize that percentage, the percentage of individuals with SmfD above the ‘usually less than 5 mf/mg skin’ warrants consideration as do challenges in monitoring participation in MDAi [60–67]. To our knowledge, levels of skin mf and ocular mf below which DEC can be administered without risk of severe Mazzotti reactions/ worsening of onchocercal eye disease and/or new ocular lesions have not been established. Furthermore, the number of individuals included in published studies is small, and we consider it insufficient to establish ‘safe’ maximum skin and ocular mf levels, in particular given inter-individual variability in the occurrence, type and severity of adverse reactions after DEC (as well as ivermectin and moxidectin) treatment. Consequently, more data are needed to determine the risk factors for the type of adverse reactions to oral DEC that could result in an unacceptable benefit-risk ratio of MDA including DEC to inform deliberations about including DEC treatment in onchocerciasis elimination strategies.

A clinical study in Ghana compared the safety and efficacy of single (*n* = 52) and three daily doses (*n* = 51) of IDA (150 µg/kg ivermectin, 6 mg/kg DEC, 400 mg albendazole) with the safety and efficacy of ivermectin plus albendazole (IA, 150 µg/kg ivermectin, 400 mg albendazole, *n* = 52) [68]. Individuals qualifying for enrolment had to meet the following criteria: (i) be among the 231 individuals who participated in a previous trial (https://www.clinicaltrials.gov/ct2/show/NCT03517462) that examined the safety and efficacy of a single dose of ivermectin for reducing skin and ocular microfilariae levels [69], (ii) had received two single doses of 150 µg/kg ivermectin > 1 year apart, the last within 1–28 weeks before IDA or IA treatment and (iii) had ≤ 3 mf/mg skin and ≤ 5 motile mf in the anterior chamber in either eye and no mf detected in the posterior segment of either eye [68]. The study found that the percentage of fertile female worms was lower (*p* = 0.004) after a single or three daily IDA doses [40/261 (15.3%) and 34/281 (12.1%), respectively] than after a single IA dose [41/180 (22.8%)]. Given the study size and inclusion criteria chosen to minimize the risk of ocular reactions, the study could not provide significant data on the safety of the proposed ‘pre-treat and treat’ MDA strategy.

We have previously reported results of our randomized, double-blind Phase 3 trial comparing the efficacy and safety of a single oral dose of 8 mg moxidectin (*n* = 978) and 150 µg/kg ivermectin (*n* = 494) in ivermectin-naïve individuals with ≥ 10 mf/mg skin with or without ocular involvement. The data showed that 1, 6, 12 and 18 months after treatment, SmfD was significantly lower among moxidectin- than ivermectin-treated participants [22, 30]. In contrast, there was no statistically significant difference in the level of live microfilariae in the anterior chamber (mfAC) 12 months after treatment among participants with > 10 mfAC pre-treatment [30].

We are here reporting the number of mfAC before treatment, 4 days and 1, 6, 12 and 18 months after treatment and their relationship to pre- and post-treatment SmfD as well as the ocular adverse events (AEs) observed within 6 months post-treatment. Our primary objective is to contribute to the evidence base available for review by WHO and countries for a decision on whether to include a ‘pretreat and treat’ strategy in control and elimination guidelines and policies for onchocerciasis and/or lymphatic filariasis in onchocerciasis co-endemic areas. While all of our participants had a pre-treatment SmfD at least twice the ‘usually less than 5 mg/skin’, the experience with severe and serious adverse reactions to ivermectin in *Loa loa* co-endemic areas has shown that decisions on control and elimination strategies need to consider risks for even a very small percentage of individuals [46, 70–73]. This need drives our emphasis on graphical presentation of individual participant data and presentation of the distribution of participants by mfAC level, which facilitate better appreciation of inter-individual differences than the standard statistical measures of variability or statistical models. Our secondary objective is to contribute to the evidence base for review by WHO and countries for decisions on whether to include moxidectin in guidelines and policies for onchocerciasis elimination.

## Methods

A detailed description of trial conduct and methods and a Consort Flow chart have been published previously [30].

### Trial registration

The study was registered on 14 November 2008 in Clinicaltrials.gov (ID: NCT00790998).

### Regulatory Agency and Ethics Committee approval and participant consent

As previously reported [22, 30], the protocol, information documents for potential participants, participant consent and assent forms and study conduct were approved by the Ghana Food and Drugs Authority and the Ghana Health Service Ethics Review Committee, the Liberia Ministry of Health and Social Welfare and the Ethics Committee of the Liberia Institute for Biomedical Research, the Ministère de la Santé Publique of the Democratic Republic of the Congo (DRC) and the Ethics Committee of the Ecole de la Santé Publique Université de Kinshasa in DRC and the WHO Ethics Review Committee.

Participants documented their informed consent or assent with parental consent to study participation through signature or thumbprint in the presence of a literate witness in their villages. This included consent to publication of summaries of the results. Given lack of consent to sharing individual participant data, many summary tables and figures are included in the manuscript or in Additional file 1.

### Overview of study conduct

This study was conducted between April 2009 and May 2012. It enrolled individuals ≥ 12 years old with at least ≥ 10 *O. volvulus* microfilariae/mg skin in four onchocerciasis-endemic areas where CDTI had not yet been initiated: Nord Kivu Province (current Zones de Santé Kalunguta and Mabalako) in DRC, Ituri province in DRC (Zone de Santé Logo in Northern Ituri, subsequently referred to as Nord-Ituri), Lofa County in Liberia (subsequently referred to as Lofa) and the Kpasa subdistrict within the Nkwanta North health district in Ghana (subsequently referred to as Nkwanta). Details on the location of the villages where participants were recruited and the prevalence of infection as determined during screening for the study have been provided previously [22].

Following randomization stratified by sex and ‘level of infection’ (< 20 mf/mg skin vs. ≥ 20 mf/mg skin), 978 participants received a single oral dose of 8 mg moxidectin and 494 participants a single oral dose of 150 µg/kg ivermectin. To ensure double blinding, each participant received four identical-looking capsules containing 2 mg moxidectin tablets, 3 mg ivermectin tablets or placebo, as required by treatment allocation and weight. The capsules for each participant had been prepared by a pharmacist not involved in participant evaluation. Participants swallowed the capsules under observation by a study team member.

Participants stayed in the research center from screening to Day 6 (± 1 Day) after treatment for follow-up examinations and daily evaluation for AEs. ‘Outpatient’ follow-up was conducted 14 days and 1, 3, 6 and 12 months after treatment (subsequently referred to as Day 14, Month 1, 3, 6 and 12, respectively). The study was initiated with 18 months post-treatment as the last follow-up timepoint. After WHO became the sole study sponsor in July 2011, resource limitations required a protocol amendment eliminating the Month 18 follow-up. This resulted in 96% of moxidectin-treated and 97% of ivermectin-treated participants with Month 12 data but only 78% of participants in both treatment arms with Month 18 data [30].

### Measurement of skin microfilariae densities

Four skin snips (one snip from each iliac crest and calf) were taken pre-treatment and at Months 1, 6, 12 and 18 and SmfD determined as described previously [21, 30].

### Ocular examinations

Ocular examinations (detailed history and questioning for symptoms, visual acuity, color vision, visual field examination with Frequency Doubling Technology (FDT) perimetry, ocular mobility, pupillary reflex examination, external ocular structures examination, anterior segment examination with a Haag Streit 900 slit-lamp, intraocular pressure and dilated fundus examination by direct and indirect ophthalmoscopy) were conducted pre-treatment as well as 3 or 4 days (subsequently referred to as Day 4 data) and at Months 1, 6, 12 and 18. mfAC and microfilariae in the cornea (mfCOR) were counted in both eyes with 10 × or 16 × magnification after participants had been sitting with their head down for at least 5 min. If considered necessary by the ophthalmologist, the head-down position for at least 5 min was repeated before counting of the microfilariae in the second eye.

### Grading of ocular AEs

Any new and clinically significant abnormality or worsening of conditions identified pre-treatment were recorded as an AE. Details of AE recording have been provided previously [30]. In brief, AEs were recorded with start and stop date and their severity graded according to the Onchocerciasis Chemotherapy Research Center (OCRC) grading scale. The scale had been developed to quantify signs and symptoms of *O. volvulus* infection caused by the immunological reaction to dying and dead microfilariae reaching the end of their natural lifespan, and their aggravation following treatment, i.e. Mazzotti reactions [74, 75]. The scale was expanded to include other types of AEs for the moxidectin Phase 2 [21, 29] and Phase 3 study [22, 30] and has been provided previously [30]. OCRC grading criteria for Mazzotti reactions differ substantially from commonly used grading criteria for similar AE and generally reflect grade for grade much less severe symptoms. For example, the National Cancer Institute criteria include need for medical intervention for grade 2 sometimes, grade 3 frequently and grade 4 nearly always. In contrast, most OCRC criteria grade 4 Mazzotti reactions require no intervention [30]. All AEs were characterized by the investigators in terms of relationship to study drug and Mazzotti reaction. Before unblinding, all AEs were reviewed centrally by one author (NOO) for characterization as Mazzotti reactions. The outcome of that central review forms the basis of the Mazzotti reactions reported here. All AE verbatims were coded using the Medical Dictionary for Regulatory Activities (MedDRA version 13.1). Mazzotti reactions were additionally coded using a Mazzotti reaction-specific dictionary since MedDRA codes the same reaction by body system, which compromises treatment comparisons [30]. Coding using the Mazzotti reaction-specific dictionary is the basis for the Mazzotti reactions reported in the manuscript. Presentation across both Mazzotti reactions and other ocular AEs in Additional file 1 is based on MedDRA coding.

### Statistical analysis

Given the focus on changes in mfAC, only the data for the 1463/1472 (99.4%) individuals treated who had both eyes evaluated throughout the study are included (Table 1). All mfAC values in the text, tables and figures are the sum of the mfAC detected across both eyes.

**Table 1:** Participants with both eyes evaluated by treatment arm, pre-treatment mfAC category and timepoint

PreTx mfAC	Moxidectin													Ivermectin												
	Any	0		1–5		6–10		11–20		21–40		> 40		Any	0		1–5		6–10		11–20		21–40		> 40	
Time	N	n	%	n	%	n	%	n	%	n	%	%	n	N	n	%	n	%	n	%	n	%	n	%	n	%
PreTx	973	596	61.3	183	18.8	59	6.1	67	6.9	52	5.3	16	1.6	490	289	59.0	96	19.6	29	5.9	45	9.2	18	3.7	13	2.7
D4	973	596	61.3	183	18.8	59	6.1	67	6.9	52	5.3	16	1.6	490	289	59.0	96	19.6	29	5.9	45	9.2	18	3.7	13	2.7
M1	967	592	61.2	181	18.7	59	6.1	67	6.9	52	5.4	16	1.7	488	287	58.8	96	19.7	29	5.9	45	9.2	18	3.7	13	2.7
M6	956	586	61.3	179	18.7	58	6.1	65	6.8	52	5.4	16	1.7	487	287	58.9	95	19.5	29	6.0	45	9.2	18	3.7	13	2.7
M12	941	574	61.0	180	19.1	57	6.1	63	6.7	51	5.4	16	1.7	476	281	59.0	93	19.5	28	5.9	43	9.0	18	3.8	13	2.7
M18	758	449	59.2	145	19.1	49	6.5	56	7.4	46	6.1	13	1.7	382	223	58.4	69	18.1	28	7.3	36	9.4	15	3.9	11	2.9

D4: Day 4 post-treatment; M1, M6, M12, M18: Month 1, 6, 12 and 18 post-treatment; mfAC: live microfilariae in the anterior chamber of the eye; PreTx: pre-treatment, percentage calculated for each timepoint by treatment arm

In recognition of the fact that as the number of microfilariae in the eyes increases beyond 10 the accuracy of the counts decreases, individual counts are presented in Figures for illustrative purposes while descriptive statistics are based on the following six mfAC categories: undetected (0), 1–5, 6–10, 11–20, 21–40, > 40. Three pre-treatment SmfD categories (10 to < 20, 20 to < 50 and ≥ 50), referred to as ‘intensity of infection’ (IoI), and three post-treatment SmfD categories (0, > 0–5, > 5) were defined.

A linear mixed model was used to evaluate the marginal means of the number of mfAC pre-treatment and on Day 4, Months 1, 6, 12 and 18. The model included treatment and sex as factors, age as adjusting variable and SmfD at baseline as covariate. Study area was included as a random effect. Treatment, sex and IoI adjusted marginal means and 95% confidence intervals (CI) by treatment were extracted from the model. A first series of models for each timepoint was run with the raw mfAC values to calculate the arithmetic adjusted marginal means. A second series of models for each timepoint was run with the log transformed mfAC values to calculate the geometric adjusted marginal means.

A linear mixed model was used to evaluate the effect of both SmfD and mfAC at the timepoint of the outcome and previous timepoints on the number of mfAC pre-treatment, Day 4, and Months 1, 6, 12 and 18. The model included treatment and sex as factors and age as adjusting variable. Study area was included as a random effect.

Generalized linear mixed models with a logit link were used to evaluate covariates and factors impacting the probability of having at least one ocular Mazzotti reaction within the 1st month after treatment and of having at least one ocular AE (whether considered Mazzotti reaction or not) within 6 months after treatment. mfAC levels were included as categorical variable (0, 1–5, 6–10 and ≥ 11) and SmfD levels as defined above. Only four mfAC level categories were considered because of the low number of individuals with mfAC ≥ 11 (*n* = 135 and *n* = 76 in the moxidectin and ivermectin treatment arm, respectively). The initial model for ocular Mazzotti reactions included treatment, age, sex, SmfD pre-treatment, mfAC levels pre-treatment and on Day 4 as fixed effects and study area as random effect. The final model included treatment, sex and mfAC levels pre-treatment. The initial model for any ocular AE during the first 6 months after treatment included treatment, age, sex, SmfD pre-treatment and at Month 1, as well as mfAC pre-treatment, on Day 4 and Month 1 as fixed effects and study area as random effect. The final model included treatment, age, sex and mfAC level at Month 1.

Analyses were conducted with SAS version 9.4 (SAS Institute, Cary, NC, USA). Figures were generated in Excel 365.

## Results

### Consort diagram and number of participants with both eyes evaluated on Day 4 and Months 1, 6, 12 and 18

The study CONSORT flow diagram has been reported previously [30].

Table 1 shows the number of individuals with data on mfAC obtained from both eyes at Day 4 and Month 1, 6, 12 and 18 by pre-treatment mfAC category and treatment arm. The maximum number of mfAC was 102 in the moxidectin and 92 in the ivermectin treatment arm. Additional file 1: Table S1 shows the number of participants by pre-treatment SmfD, mfAC category and sex.

### Number of live microfilariae in the anterior chambers on Day 4 and Months 1, 6, 12 and 18

**Fig. 1:**
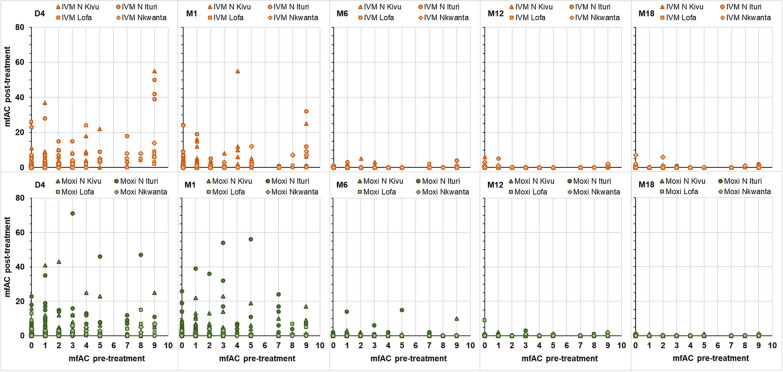
mfAC 4 days, 1, 6, 12 and 18 months post-treatment relative to pre-treatment mfAC among participants with < 10 mfAC pre-treatment. *x-axis* mfAC levels pre-treatment, *y-axis* mfAC levels on Day 4 (D4 1st column), Month 1 (M1 2nd column), Month 6 (M6, 3rd column), Month 12 (M12 4th column) and Month 18 (M18, 5th column) post-treatment with ivermectin (IVM, upper row, orange symbols) or moxidectin (Moxi, lower row, green symbols). Data from participants from Nord Kivu (∆), Nord Ituri (○), Lofa County (□) and Nkwanta district (◊). mfAC live microfilariae in the anterior chambers

**Fig. 2:**
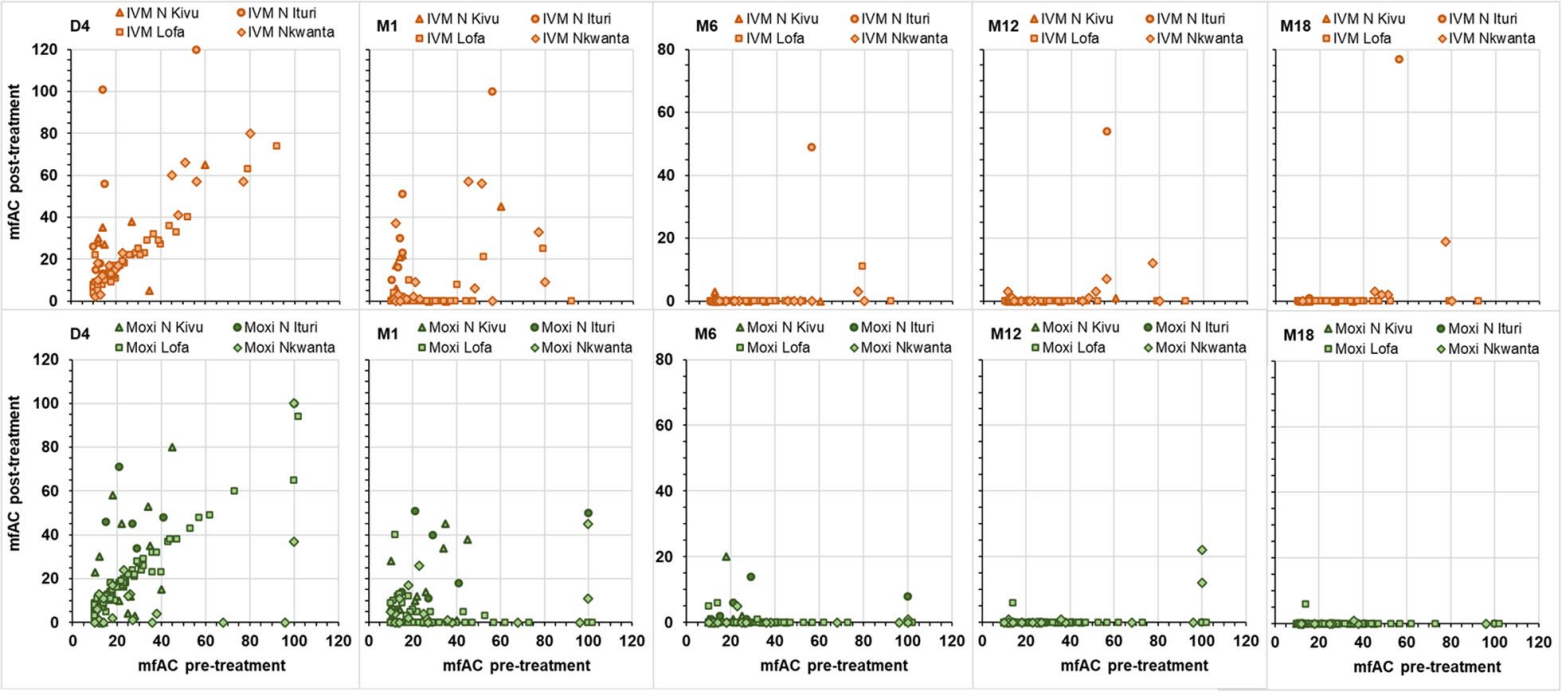
mfAC 4 days, 1, 6, 12 and 18 months post-treatment relative to pre-treatment mfAC among participants with ≥ 10 mfAC pre-treatment. *x-axis* mfAC levels pre-treatment, *y-axis *mfAC levels on Day 4 (D4 1st column), Month 1 (M1 2nd column), Month 6 (M6, 3rd column), Month 12 (M12 4th column) and Month 18 (M18, 5th column) post-treatment with ivermectin (IVM, upper row, orange symbols) or moxidectin (Moxi, lower row, green symbols). Data from participants from Nord Kivu (∆), Nord Ituri (○), Lofa County (□) and Nkwanta district (◊). mfAC live microfilariae in the anterior chambers

Figures 1 and 2 show the mfAC levels on Day 4 and Month 1, 6, 12 and 18 relative to pre-treatment levels for each participant and illustrate the inter-individual variability in the mfAC level change after treatment.

**Fig. 3:**
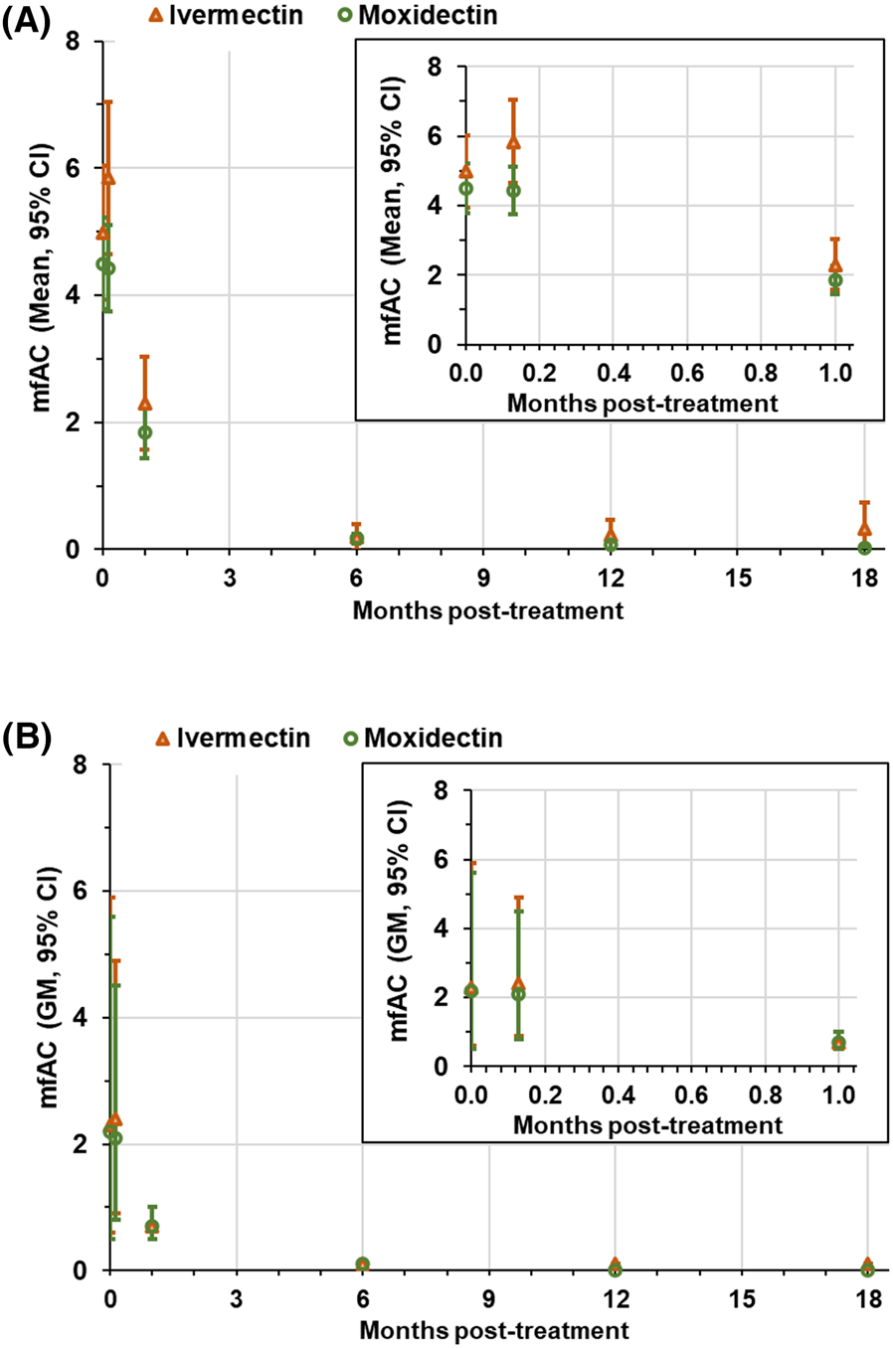
Arithmetic mean (**A**) and linear model derived adjusted geometric mean (**B**) mfAC pre-treatment (month 0), 4 days, 1, 6, 12 and 18 months post-treatment in the moxidectin and ivermectin treatment arm. Orange triangle: data in the ivermectin treatment arm, green circles: data in the moxidectin treatment arm. Mean: arithmetic mean; GM: geometric mean; CI: confidence interval; mfAC, live microfilariae in the anterior chambers. The inserts provide an enlargement of the data obtained pre-treatment (Month 0) and 4 days and 1 month after treatment

Figure 3 shows the arithmetic means and the linear model derived adjusted geometric mean mfAC pre-treatment and on Day 4 and Month 1, 6, 12 and 18.

Increases in the number of mfAC detected sufficient to change the mfAC category occurred from pre-treatment to Day 4 and from pre-treatment to Month 1, respectively, in 21% and 16% in the moxidectin treatment arm and 19% and 15% in the ivermectin treatment arm. Furthermore, in 11% of participants in both treatment arms mfAC levels increased from Day 4 to Month 1 (Table 2). This resulted in maximum mfAC levels of up to 23 and 26 on Day 4 and of up to 26 and 24 at Month 1 among participants with undetectable mfAC levels pre-treatment in the moxidectin and ivermectin treatment arm, respectively (Additional file 1: Table S2). The mean, standard deviation, minimum and maximum mfAC levels pre-treatment, on Day 4 and at Month 1 for all participants with mfAC increases from pre-treatment to Day 4 or Month 1 and from Day 4 to Month 1 are provided in Additional file 1: Table S2.

**Table 2:** Number (%) of participants with mfAC number increases from pre-treatment to Day 4 or Month 1 and from Day 4 to Month 1 resulting in mfAC levels in a higher mfAC category

mfAC category	Moxidectin													Ivermectin													
	Nf	1–5 mfAC		6–10 mfAC			11–20 mfAC		21–40 mfAC		> 40		Any mfAC increase	Nf		1–5 mfAC		6–10 mfAC		11–20 mfAC		21–40 mfAC		> 40 mfAC		Any mfAC increase	
		n	%	n	%	n		%	n	%	n	%	n	%		N	%	n	%	n	%	n	%	n	%	n	%
**PreTx**		**Day 4**														**Day 4**											
**0**	596	135	22.7	11	1.8	3		0.5	1	0.2			150	25.2	289	49	17.0	4	1.4	1	0.3	2	0.7			56	19.4
**1–5**	183			16	8.7	13		7.1	3	1.6	4	2.2	36	19.7	96			15	15.6	3	3.1	4	4.2			22	22.9
**6–10**	59					3		5.1	4	6.8	2	3.4	9	15.3	29					2	6.9	2	6.9	3	10.3	7	24.1
**11–20**	67								1	1.5	2	3.0	3	4.5	45							5	11.1	2	4.4	7	15.6
**21–40**	52										4	7.7	4	7.7	18												
**Sum**	957	135	14.1	27	2.8	19		2.0	9	0.9	12	1.3	202	21.1	477	49	10.3	19	4.0	6	1.3	13	2.7	5	1.0	92	19.3
**PreTx**		**Month 1**														**Month 1**											
**0**	592	113	19.1	5	0.8	2		0.3	1	0.2			121	20.4	287	45	15.7	6	2.1			1	0.3			52	18.1
**1–5**	181			9	5.0	9		5.0	5	2.8	2	1.1	25	13.8	96			3	3.1	6	6.3			1	1.0	10	10.4
**6–10**	59					4		6.8	3	5.1	1	1.7	8	13.6	29					1	3.4	2	6.9			3	10.3
**11–20**	67								1	1.5			1	1.5	45							5	11.1	1	2.2	6	13.3
**21–40**	52										2	3.8	2	3.8	18												
**Sum**	951	113	11.9	14	1.5	15		1.6	10	1.1	5	0.5	157	16.5	475	45	9.5	9	1.9	7	1.5	8	1.7	2	0.4	71	14.9
**Day 4**		**Month 1**														**Month 1**											
**0**	533	68	12.8	9	1.7	2		0.4					79	14.8	263	37	14.1	4	1.5							41	15.6
**1–5**	237			4	1.7	4		1.7	3	1.3	1	0.4	12	5.1	105			3	2.9	1	1.0			1	1.0	5	4.8
**6–10**	71					2		2.8	3	4.2			5	7.0	42					2	4.8					2	4.8
**11–20**	72								2	2.8			2	2.8	34							2	5.9			2	5.9
**21–40**	32										2	6.3	2	6.3	29												
**Sum**	945	68	7.2	13	1.4	8		0.8	8	0.8	3	0.3	100	10.6	473	37	7.8	7	1.5	3	0.6	2	0.4	1	0.2	50	10.6

mfAC: live microfilariae in the anterior chamber; PreTx: pre-treatment; Nf: number of participants with data at relevant follow-up timepoints; % calculated based on Nf

Additional file 1: Table S3 shows the distribution of participants by mfAC category at Day 4 and Month 1 relative to their distribution pre-treatment and on Day 4 resulting from increases in mfAC levels in some participants and decreases or changes not affecting the mfAC category in others. Across all participants, 54.9% and 71.5% of moxidectin-treated and 54.1% and 71.1% of ivermectin-treated participants had no mfAC detected on Day 4 and Month 1, respectively. mfAC levels > 10 were detected on Day 4 and Month 1 in 13.1% and 4.9% of moxidectin-treated and 15.9% and 5.3% of ivermectin-treated participants, respectively.

At Month 6, 12 and 18, the percentage of participants with 0 mfAC and 1–5 mfAC detected was 94.8% and 4.3%, 98.1% and 1.5%, and 98.7% and 1.2%, respectively, in the moxidectin treatment arm and 95.5% and 4.1%, 95.6% and 3.6%, and 95.8% and 3.1% in the ivermectin treatment arm. With two exceptions, the remaining participants had mfAC levels in the 6–10 or 11–20 mfAC category. Maximum mfAC levels at Month 6, 12 and 18 were 20, 12 and 6 in the moxidectin treatment arm and 11, 12 and 19 in the ivermectin treatment arm, respectively (Table 3). One participant from Nord Ituri treated with ivermectin had mfAC levels of 56, 120, 100, 49, 54 and 77 pre-treatment and on Day 4, Month 1, 6, 12 and 18, respectively, with SmfD levels of 53, 53, 32, 49 and 72 pre-treatment and at Month 1, 6, 12 and 18, respectively, qualifying for suboptimal microfilariae response and suboptimal response [22]. The Mazzotti reactions the participant experienced starting 1 day after ivermectin administration (dizziness, headache, facial oedema) suggest that pharmacodynamically sufficient ivermectin was absorbed even though the participant was recorded as having grade 1 diarrhoea from the day before to 2 days after ivermectin administration. One participant from Nkwanta treated with moxidectin had mfAC levels of 100, 100, 45, 0 and 22 pre-treatment and on Day 4, Month 1, 6 and 12, respectively, with SmfD levels of 44, 0, 0 and 0.4 pre-treatment and at Month 1, 6 and 12, respectively. That participant did not have a Month 18 evaluation because of the protocol amendment abolishing the Month 18 visit.

**Table 3:** Number (%) of participants by mfAC category at Months 6, 12 and 18 by pre-treatment mfAC category and treatment arm

PreTx	Moxidectin													Ivermectin												
mfAC	Any mfAC	0 mfAC		1–5 mfAC		6–10 mfAC		11–20 mfAC		21–40 mfAC		> 40 mfAC		Any mfAC	0 mfAC		1–5 mfAC		6–10 mfAC		11–20 mfAC		21–40 mfAC		> 40 mfAC	
	N	n	%	n	%	n	%	n	%	n	%	n	%	N	n	%	n	%	n	%	n	%	n	%	n	%
		**Month 6**													**Month 6**											
0	586	571	97.4	15	2.6		0.0							287	279	97.2	8	2.8								
1–5	179	164	91.6	12	6.7	1	0.6	2	1.1					95	89	93.7	6	6.3								
6–10	58	51	87.9	6	10.3	1	1.7							29	26	89.7	3	10.3								
11–20	65	61	93.8	2	3.1	1	1.5	1	1.5					45	43	95.6	2	4.4								
21–40	52	45	86.5	5	9.6	1	1.9	1	1.9					18	18	100.0										
> 40	16	14	87.5	1	6.3	1	6.3							13	10	76.9	1	7.7			1	7.7			1	7.7
All	956	906	94.8	41	4.3	5	0.5	4	0.4					487	465	95.5	20	4.1			1	0.2			1	0.2
		**Month 12**													**Month 12**											
0	574	570	99.3	3	0.5	1	0.2							281	276	98.2	4	1.4	1	0.4						
1–5	180	175	97.2	5	2.8									93	91	97.8	2	2.2								
6–10	57	53	93.0	4	7.0									28	26	92.9	2	7.1								
11–20	63	61	96.8	1	1.6	1	1.6							43	37	86.0	6	14.0								
21–40	51	50	98.0	1	2.0									18	18	100.0		0.0								
> 40	16	14	87.5		0.0			1	6.3	1	6.3			13	7	53.8	3	23.1	1	7.7	1	7.7			1	7.7
All	941	923	98.1	14	1.5	2	0.2	1	0.1	1	0.1			476	455	95.6	17	3.6	2	0.4	1	0.2			1	0.2
		**Month 18**													**Month 18**											
0	449	444	98.9	5	1.1									223	220	98.7	2	0.9	1	042						
1–5	145	143	98.6	2	1.4									69	65	94.2	3	4.3	1	1.4						
6–10	49	48	98.0	1	2.0									28	25	89.3	3	10.7								
11–20	56	55	98.2			1	1.8							36	35	97.2	1	2.8								
21–40	46	45	97.8	1	2.2									15	15	100.0		0.0								
> 40	13	13	100.0											11	6	54.5	3	27.3			1	9.1			1	9.1
All	758	748	98.7	9	1.2	1	0.1							382	366	95.8	12	3.1	2	0.5	1	0.3			1	0.3

mfAC: live microfilariae in the anterior chambers; % calculated based on all participants with data in the specified PreTx mfAC category (N)

### Pre- and post-treatment mfAC levels relative to pre-treatment SmfD (IoI)

Additional file 1: Figs. S1 and S2 show for participants with < 10 mfAC and ≥ 10 mfAC pre-treatment, respectively, the mfAC counts before and on Days 4 and Month 1, 6, 12 and 18 by IoI and illustrate the inter-individual variability in pre-treatment as well as post-treatment mfAC relative to IoI.

Table 4 provides the corresponding distribution of participants in the defined pre-treatment mfAC categories by IoI. The data indicate that as the IoI increases the percentage of individuals with undetected mfAC decreases. Across both treatment arms, this percentage was 70.3%, 63.3% and 45.6% for participants with IoI ≤ 20 SmfD, 20– < 50 SmfD and ≥ 50 SmfD, respectively. The percentage with mfAC pre-treatment ≥ 40 was 1.9%, 1.6% and 2.7%, respectively. Additional file 1: Table S2 provides these percentages for all mfAC categories. Table 5 presents the distribution by mfAC categories at Day 4 and Month 1 for the participants with an increase in mfAC levels from pre-treatment to Day 4 and Month 1 and from Day 4 to Month 1 that resulted in a higher mfAC category by pre-treatment SmfD category. The data suggest that a higher pre-treatment SmfD is associated with a higher percentage of individuals who experienced a transient increase in mfAC from pre-treatment to Day 4 and Month 1.

**Table 4:** Number (%) of participants by mfAC category pre-treatment and on Day 4 and Month 1, 6, 12 and 18 by intensity of infection

IoI	Moxidectin													Ivermectin												
	All	0 mfAC		1–5 mfAC		6–10 mfAC		11–20 mfAC		21–40 mfAC		> 40 mfAC		All	0 mfAC		1–5 mfAC		6–10 mfAC		11–20 mfAC		21–40 mfAC		> 40 mfAC	
	N	n	%	N	%	n	%	n	%	n	%	n	%	N	N	%	n	%	n	%	n	%	n	%	n	%
	**Pre-treatment**																									
< 20	**279**	197	70.6	27	9.7	14	5.0	21	7.5	18	6.5	2	0.7	**148**	103	69.6	16	10.8	5	3.4	12	8.1	6	4.1	6	4.1
20- < 50	**454**	299	65.9	77	17.0	25	5.5	25	5.5	20	4.4	8	1.8	**181**	103	56.9	43	23.8	12	6.6	15	8.3	6	3.3	2	1.1
≥ 50	**240**	100	41.7	79	32.9	20	8.3	21	8.8	14	5.8	6	2.5	**161**	83	51.6	37	23.0	12	7.5	18	11.2	6	3.7	5	3.1
All	**973**	596	61.3	183	18.8	59	6.1	67	6.9	52	5.3	16	1.6	**490**	289	59.0	96	19.6	29	5.9	45	9.2	18	3.7	13	2.7
	**Day 4**																									
< 20	**279**	170	60.9	59	21.1	17	6.1	21	7.5	8	2.9	4	1.4	**148**	98	66.2	18	12.2	10	6.8	8	5.4	10	6.8	4	2.7
20- < 50	**454**	259	57.0	113	24.9	30	6.6	33	7.3	12	2.6	7	1.5	**181**	108	59.7	39	21.5	13	7.2	13	7.2	7	3.9	1	0.6
≥ 50	**240**	105	43.8	68	28.3	25	10.4	19	7.9	12	5.0	11	4.6	**161**	59	36.6	48	29.8	19	11.8	13	8.1	12	7.5	10	6.2
All	**973**	534	54.9	240	24.7	72	7.4	73	7.5	32	3.3	22	2.3	**490**	265	54.1	105	21.4	42	8.6	34	6.9	29	5.9	15	3.1
	**Month 1**																									
< 20	**278**	233	83.8	39	14.0	2	0.7	2	0.7	2	0.7			**148**	120	81.1	18	12.2	4	2.7	2	1.4	4	2.7		
20- < 50	**450**	332	73.8	86	19.1	16	3.6	13	2.9	2	0.4	1	0.2	**181**	136	75.1	39	21.5	4	2.2	1	0.6	1	0.6		
≥ 50	**239**	126	52.7	77	32.2	9	3.8	11	4.6	10	4.2	6	2.5	**159**	91	57.2	38	23.9	12	7.5	6	3.8	6	3.8	6	3.8
All	**967**	691	71.5	202	20.9	27	2.8	26	2.7	14	1.4	7	0.7	**488**	347	71.1	95	19.5	20	4.1	9	1.8	11	2.3	6	1.2
	**Month 6**																									
< 20	**273**	269	98.5	4	1.5									**148**	144	97.3	3	2.0			1	0.7				
20- < 50	**446**	424	95.1	20	4.5	2	0.4							**179**	170	95.0	9	5.0								
≥ 50	**237**	213	89.9	17	7.2	3	1.3	4	1.7					**160**	151	94.4	8	5.0							1	0.6
All	**956**	906	94.8	41	4.3	5	0.5	4	0.4					**487**	465	95.5	20	4.1			1	0.2			1	0.2
	**Month 12**																									
< 20	**268**	265	98.9	2	0.7	1	0.4							**146**	140	95.9	4	2.7	1	0.7	1	0.7				
20- < 50	**437**	426	97.5	9	2.1	1	0.2			1	0.2			**175**	172	98.3	3	1.7								
≥ 50	**236**	232	98.3	3	1.3			1	0.4					**155**	143	92.3	10	6.5	1	0.6					1	0.6
All	**941**	923	98.1	14	1.5	2	0.2	1	0.1	1	0.1			**476**	455	95.6	17	3.6	2	0.4	1	0.2			1	0.2
	**Month 18**																									
< 20	**224**	223	99.6	1	0.4									**115**	111	96.5	2	1.7	1	0.9	1	0.9				
20- < 50	**334**	327	97.9	6	1.8	1	0.3							**138**	137	99.3	1	0.7								
≥ 50	**200**	198	99.0	2	1.0		0.0							**129**	118	91.5	9	7.0	1	0.8					1	0.8
All	**758**	748	98.7	9	1.2	1	0.1							**382**	366	95.8	12	3.1	2	0.5	1	0.3			1	0.3

PreTx pre-treatment, IoI pre-treatment skin microfilariae density (microfilariae/mg skin)

**Table 5:** Number (%) of participants by mfAC category on Day 4 and Month 1 and pre-treatment IoI for participants with mfAC number increases from pre-treatment to Day 4 or Month 1 and from Day 4 to Month 1 resulting in mfAC levels in a higher mfAC category

IoI	Moxidectin													Ivermectin												
		1–5		6–10		11–20		21–40		> 40		Any increase			1–5		6–10		11–20		21–40		> 40		Any increase	
	N	n	%	n	%	n	%	n	%	n	%	n	%	N	n	%	n	%	n	%	n	%	n	%	N	%
	Day 4 mfAC category of participants with mfAC increase from preTx to Day 4																									
< 20	279	38	13.62	1	0.36	1	0.36	1	0.36	2	0.72	43	15.41	148	38	13.62	1	0.36	1	0.36	1	0.36	2	0.72	43	15.41
20- < 50	454	68	14.98	13	2.86	7	1.54	2	0.44	2	0.44	92	20.26	181	68	14.98	13	2.86	7	1.54	2	0.44	2	0.44	92	20.26
≥ 50	240	29	12.08	13	5.42	11	4.58	6	2.5	8	3.33	67	27.92	161	29	12.08	13	5.42	11	4.58	6	2.5	8	3.33	67	27.92
All	973	135	13.87	27	2.77	19	1.95	9	0.92	12	1.23	202	20.76	490	135	13.87	27	2.77	19	1.95	9	0.92	12	1.23	202	20.76
	Month 1 mfAC category for participants with mfAC increase from preTx to Month 1																									
< 20	278	24	8.63		0	1	0.36		0		0	25	8.99	148	10	6.76	1	0.68	2	1.35	2	1.35		0	15	10.14
20- < 50	450	56	12.44	8	1.78	8	1.78	1	0.22		0	73	16.22	181	16	8.84		0	1	0.55		0		0	17	9.39
≥ 50	239	33	13.81	6	2.51	6	2.51	9	3.77	5	2.09	59	24.69	159	19	11.95	8	5.03	4	2.52	6	3.77	2	1.26	39	24.53
All	967	113	11.69	14	1.45	15	1.55	10	1.03	5	0.52	157	16.24	488	45	9.22	9	1.84	7	1.43	8	1.64	2	0.41	71	14.55
	Month 1 mfAC category for participants with mfAC increase from Day 4 to Month 1																									
< 20	278	13	4.68		0	1	0.36		0		0	14	5.04	148	9	6.08	1	0.68	1	0.68	1	0.68		0	12	8.11
20- < 50	450	32	7.11	9	2	3	0.67	1	0.22		0	45	10.00	181	20	11.05		0	1	0.55		0		0	21	11.60
≥ 50	239	23	9.62	4	1.67	4	1.67	7	2.93	3	1.26	41	17.15	159	8	5.03	6	3.77	1	0.63	1	0.63	1	0.63	17	10.69
All	967	68	7.03	13	1.34	8	0.83	8	0.83	3	0.31	100	10.34	488	37	7.58	7	1.43	3	0.61	2	0.41	1	0.2	50	10.25

mfAC: live microfilariae in the anterior chamber; PreTx: pre-treatment; N: number of participants with mfAC data at Day 4 and Month 1, respectively; % calculated based on total number of participants with mfAC data on Day 4 or Month 1 within each IoI category

### Post-treatment mfAC levels relative to post-treatment SmfD

Table 6 shows the distribution of participants in each treatment arm by mfAC category and SmfD category at Month 6, 12 and 18. At Month 6, 12 and 18 after ivermectin treatment, mfAC were undetectable in all of the 54, 24 and 15 individuals with undetectable SmfD, respectively, while mfAC were undetectable in 91.5%, 92.9% and 94.1% of the 94, 240 and 237 individuals with > 5 SmfD, respectively. At Month 6, 12 and 18 after moxidectin treatment, the percentage of participants with undetectable mfAC was 94.9%, 99.5% and 99.5% among the 830, 434 and 211 participants with undetectable SmfD, respectively. No moxidectin-treated participant had > 5 SmfD at Month 6. At Month 12 and 18 after moxidectin treatment, mfAC were not detected in 90.5% and 95.9% of the 74 and 170 individuals with > 5 SmfD, respectively. Additional file 1: Figs. S3 and S4 show the data for each individual participant for those with < 10 mfAC and those with ≥ 10 mfAC pre-treatment, respectively. They illustrate not only the inter-individual variability in the change in mfAC relative to the change in SmfD but also that mfAC reduction can be slower than SmfD reduction [30].

**Table 6:** Number (%) of participants by mfAC category at Month 6, 12 and 18 by post-treatment SmfD and treatment

SmfD	Moxidectin													Ivermectin													
	Any	0		1–5		6–10		11–20		21–40		> 40		Any		0	1–5		6–10		11–20		21–40			> 40	
	N (%)^1^	n	%^2^	n	%	n	%	n	%	n	%	n	%	N (%)^1^	n	%^2^	n	%	n	%	n	%	n	%	n		%
Month 6	Month 6																										
0	875 (91.5)	830	94.9	37	4.2	4	0.5	4	0.5					54 (11.1)	54	100											
> 0–5	81 (8.5)	76	93.8	4	4.9	1	1.2							339 (69.6)	325	95.9	13	3.8			1	0.3					
> 5	0 (0)													94 (19.3)^4^	86	91.5	7	7.4							1		1.1
All	956 (100)	906	94.8	41	4.3	5	0.5	4	0.4					487 (100)	465	95.5	20	4.1			1	0.2			1		0.2
Month 12	Month 12																										
0	436 (46.3)	434	99.5	2	0.5									24 (5.0)	24	100											
> 0–5	431 (45.8)	422	97.9	6	1.4	2	0.5			1	0.2			212 (44.5)	208	98.1	2	0.9	1	0.5	1	0.5					
> 5	74 (7.9)^3^	67	90.5	6	8.1			1	1.4					240 (50.4)^4^	223	92.9	15	6.3	1	0.4					1		0.4
All	941 (100)	923	98.1	14	1.5	2	0.2	1	0.1	1	0.1			476 (100)	455	95.6	17	3.6	2	0.4	1	0.2			1		0.2
Month 18	Month 18																										
0	212 (28.0)	211	99.5	1	0.5									15 (3.9)	15	100											
> 0–5	376 (49.6)	374	99.5	1	0.3	1	0.3							130 (34.0)	128	98.5	2	1.5									
> 5	170 (22.4)^3^	163	95.9	7	4.1									237 (62.0)^4^	223	94.1	10	4.2	2	0.8	1	0.4			1		0.4
All	758 (100)	748	98.7	9	1.2	1	0.1							382 (100)	366	95.8	12	3.1	2	0.5	1	0.3			1		0.3

^1^% calculated across SmfD categories, ^2^% calculated by SmfD category. ^3^ maximum SmfD of 27.9 and 70.5 at Month 12, respectively, ^4^maximum SmfD 43.1, 76.5 and 101.3 at Month 6, 12 and 18, respectively (for further details see Additional file 1: Fig S3 and Fig S4)

### Participant characteristics impacting mfAC levels before and after treatment

As previously reported for mfAC levels at Month 12 in participants with at least 10 mfAC [30], there was no statistically significant difference at any timepoint between the mfAC levels in the moxidectin and the ivermectin treatment arm. Age and sex were not a significant covariate and factor, respectively. Table 7 provides an overview of the impact on the mfAC levels at a particular timepoint of the other covariates (SmfD before or at that timepoint and prior mfAC levels) emerging from the linear model. For all post-treatment timepoints, the mfAC levels were statistically significantly impacted by at least two of these covariates. Additional file 1: Table S4 shows the full output.

**Table 7:** Impact of SmfD and mfAC levels up to the relevant timepoint on mfAC levels pre-treatment and at different timepoints post-treatment

mfAC at time	Covariates in the final model	*p* value ≤ 0.05	*p* value > 0.5
PreTx	SmfD preTx	< 0.0001	
Day 4	SmfD preTx	0.0006	
	mfAC preTx	< 0.0001	
Month 1	SmfD preTx	< 0.0001	
	SmfD Month 1	0.0058	
	mfAC preTx		0.1469
	mfAC Day 4	< 0.0001	
Month 6	SmfD preTx		0.9828
	SmfD Month 1	0.0002	
	SmfD Month 6		0.1561
	mfAC preTx		0.6150
	mfAC Day 4	0.0419	
	mfAC Month 1	< 0.0001	
Month 12	SmfD preTx	0.0015	
	SmfD Month 1		0.0544
	SmfD Month 6		0.8066
	SmfD Month 12	0.001	
	mfAC preTx	0.0015	
	mfAC Day 4		0.2651
	mfAC Month 1	< 0.0001	
	mfAC Month 6	< 0.0001	
Month 18	SmfD preTx		0.1763
	SmfD Month 1		0.9814
	SmfD Month 6		0.3455
	SmfD Month 12		0.2232
	SmfD Month 18		0.7997
	mfAC preTx		0.2282
	mfAC Day 4		0.6282
	mfAC Month 1		0.8748
	mfAC Month 6	0.0001	
	mfAC Month 12	< 0.0001	

mfAC: live microfilariae in the anterior chambers; SmfD: skin microfilariae density (microfilariae/mg skin)

### Microfilariae in the cornea

Live microfilariae in the cornea were detected pre-treatment in the majority of participants from Lofa (count range 3–42) but in only few participants in the other study areas. Similarly, dead microfilariae in the cornea were more frequently seen in participants from Lofa (count range 2–25) than in participants from the other study areas (count range 0–18). The reason for this difference is unknown. At Month 6, 12 and 18, live microfilariae in the cornea were detected only in five (count range 1–9), zero and one (count = 2) participants from Lofa, respectively. Dead microfilariae in the cornea at Month 6, 12 and 18 were found in one (count = 6), zero and zero participants from Nord Kivu, two (counts = 2), zero and one (count = 1) participants from Nord-Ituri, 18 (count range 1–29), two (count = 1) and one (count = 2) participant from Lofa and in zero, one (count = 1) and zero participant from Nkwanta, respectively.

### Ocular Mazzotti reactions and other ocular AEs

As reference for the ocular Mazzotti reactions and other post-treatment ocular adverse events, Additional file 1: Table S5 provides the ocular medical history the investigators considered relevant and entered into the data base by study area.

As previously reported across all participants treated [30], a higher percentage of participants treated with moxidectin (121/973, 12.4%) than with ivermectin (50/490, 10.2%) had at least one ocular Mazzotti reaction, but the type of reactions did not differ (Table 8). Among those with ocular Mazzotti reactions, a higher percentage of moxidectin treated (26/121, 21.5%) than ivermectin treated (6/50, 12%) participants had more than one ocular Mazzotti reaction, but there was no trend towards higher severity of the ocular Mazzotti reactions after moxidectin than ivermectin treatment (Table 9). The vast majority of Mazzotti reactions occurred between the day of treatment up to the 2nd day after treatment with a trend towards earlier start after moxidectin than after ivermectin treatment (Table 10), a trend also seen for non-ocular Mazzotti reactions.

**Table 8:** Participants with ocular Mazzotti reactions starting within 1 Month of treatment among participants with both eyes evaluated by treatment

	Moxidectin	N = 973^1^	Ivermectin	N = 490^1^
*Mazzotti sign/ symptom*	n	%	n	%
Eye pruritus	45	4.62	12	2.45
Conjunctivitis	41	4.21	15	3.06
Eye pain	29	2.98	8	1.63
Eyelid oedema	20	2.06	5	1.02
Ocular discomfort^2^	9	0.92	5	1.02
Tearing/watery eyes	7	0.72	7	1.43
Blurred vision	3	0.31	3	0.61
Photophobia	2	0.21	1	0.20
Peripheral sensory phenomena^3^	1	0.10	2	0.41
Visual acuity^4^	1	0.10		0
Total number of participants with at least 1 ocular Mazzotti reaction	121	12.4	50	10.2
Total number of Mazzotti reactions	158		58	

^1^Among the nine participants who did not have both eyes evaluated at all visits they attended, one had an ocular Mazzotti reaction (pain in right eye, 26 days after ivermectin treatment)

^2^Verbatims for ‘Ocular discomfort’ included in the moxidectin arm: ‘ocular discomfort’ (n = 5), ‘foreign body sensation in eyes’ (*n* = 3) and ‘sandy sensation of eyes’ (*n* = 1) and in the ivermectin arm ‘ocular discomfort’ (*n* = 2), ‘foreign body sensation in eyes’ (*n* = 2) and ‘cumbersome upper eyelids’ (*n* = 1)

^3^Verbatims for ‘peripheral sensory phenomena’ included ‘heavy sensation of the eyelids’ (moxidectin, duration 1 day), ‘sandy sensation in eyes’ (ivermectin, duration 7 days) and ‘burning sensation in the eyes’ (ivermectin, duration 132 days)

^4^Verbatim: ‘reduced vision’ (moxidectin, duration 1 day)

**Table 9:** Participants with ocular Mazzotti reactions starting within 1 Month of treatment by severity and treatment among participants with both eyes evaluated

Mazzotti sign/symptom	Grade	Moxidectin	N = 973	Ivermectin	N = 490
		n	%	n	%
Eye pruritus	1	35	3.6	7	1.4
	2	8	0.8	4	0.8
	3	2^1^	0.2	1	0.2
Conjunctivitis	1	38^2^	3.9	14	2.9
	2	2	0.2		
	3	1	0.10	1	0.2
Eye pain	1	25	2.6	8	1.6
	2	4	0.4		
Eyelid oedema	1	18	1.8	5	1.0
	2	2	0.2		0.0
Blurred vision	1	3	0.3	2	0.4
	2			1	0.2
Ocular discomfort	1	8	0.8	4^3^	0.8
	2	1	0.1	1	0.2
Tearing/watery eyes	1	6^4^	0.6	5^5^	1.0
	2	1^4^	0.1	2	0.4
Photophobia	1	2	0.2	1	0.2
Peripheral sensory phenomena	1	1	0.1	2	0.4
Visual acuity^6^	1	1	0.1		

^1^Moxidectin treatment: One participant had eye pruritus grade 3 starting 1 day after treatment which lessened to grade 2 on day 2 and resolved 2 days later. One participant had eye pruritus grade 3 starting on the day of treatment which resolved 2 days later

^2^ Moxidectin treatment: One participant had conjunctivitis grade 1 starting on the day of treatment, which worsened to grade 3 on the day after treatment before resolving 3 days later

^3^Ivermectin treatment: One participant had ocular discomfort grade 1 starting on the day of treatment which resolved the next day, reappeared on Day 3 and resolved the next day

^4^Moxidectin treatment: One participant had watery eyes starting on the day of treatment which resolved 2 days later and reappeared on Day 11 to resolved 6 days later. One participant had grade 2 watery eyes starting on the day of treatment which resolved the next day and experienced grade 1 watery eyes starting 8 days after treatment which resolved 43 days later

^5^Ivermectin treatment: One participant had eye pruritus grade 3 starting on the day of treatment which resolved 2 days later

^6^Moxidectin treatment: One participant complained about ‘reduced vision’ starting on Day 2 after treatment which resolved on Day 3 after treatment

**Table 10:** Participants with ocular Mazzotti reactions starting within 1 Month of treatment by start day and treatment among participants with both eyes evaluated

Mazzotti sign/ symptom	Start after treatment (days)	Moxidectin			Ivermectin		
		n	% of N (973)	% of participants with that ocular Mazzotti reaction	n	% of N (490)	% of participants with that ocular Mazzotti reaction
Eye pruritus	0	21	2.2	46.7	3	0.6	25.0
	1	17	1.7	37.8	6	1.2	50.0
	2	2	0.2	4.4	1	0.2	8.3
	3				1	0.2	8.3
	4	1	0.1	2.2			
	8	1	0.1	2.2			
	11				1	0.2	8.3
	15	1	0.1	2.2			
	21	2	0.2	4.4			
Conjunctivitis	0	24	2.5	58.5	3	0.6	20.0
	1	11	1.1	26.8	4	0.8	26.7
	2	3	0.3	7.3	8	1.6	53.3
	3	2	0.2	4.9			
	5	1	0.1	2.4			
Eye pain	0	8	0.8	27.6	1	0.2	12.5
	1	14	1.4	48.3	2	0.4	25.0
	2	2	0.2	6.9	5	1.0	62.5
	3	2	0.2	6.9			
	4	2	0.2	6.9			
	10	1	0.1	3.5			
Eyelid oedema	0	8	0.8	40.0			
	1	7	0.7	40.0	4	0.8	80.0
	2	4	0.4	40.0			
	3	1	0.1	40.0	1	0.2	20.0
Ocular discomfort	0	7	0.7	77.8	1	0.2	20.0
	1	1	0.1	11.1	1	0.2	20.0
	3	1	0.1	11.1	2	0.4	40.0
	9				1	0.2	20.0
Tearing/watery eyes	0	3	0.3	0.4	2	0.4	28.6
	1	1	0.1	0.1	3	0.6	42.9
	2			0.00	2	0.4	28.6
	4	1	0.1	0.1			
	8	1	0.1	0.1			
	11	1	0.1	0.1			
Blurred vision	0	2	0.2	66.7			
	1				3	0.6	100
	21	1	0.1	33.3			
Photophobia	1				1	0.2	
	2	2	0.2				
Peripheral sensory phenomena	0	1	0.1		1	0.2	
	1				1	0.2	
Visual acuity	2	1	0.1				

The logistic model identified women as at higher risk for ocular Mazzotti reactions (OR 1.537, 95% CI 1.096–2.157, *p* = 0.0128) and detected pre-treatment mfAC levels as impacting the risk for ocular Mazzotti reactions (OR for 0 mfAC: > 10 mfAC 2.704 with 95% CI 1.272–5.749, *p* = 0.0098, *p* = 0.0608 for analysis across all mfAC level categories). The OR for treatment with moxidectin: ivermectin was 1:0.746 (95% CI 0.519–1.072, *p* = 0.1127). Additional file 1: Table S6 shows the output of the final model.

At least one ocular AE (i.e. considered Mazzotti reaction or not) was recorded for 223/973 (22.9%) and 82/490 (16.7%) of participants to Month 1 and for 92/973 (9.5%) and 43/490 (8.8%) of participants from Month 1 to Month 6 after treatment with moxidectin and ivermectin, respectively. An overview of participant incidence and total number of ocular AEs is provided in Additional file 1: Tables S7 and S8. Grade 3 ocular AEs not considered a Mazzotti reaction in the moxidectin treatment arm were eye pruritus in three participants (starting 64, 86 and 159 days after treatment, respectively), eye pain starting 173 days after treatment and cyclitis starting 174 days after treatment. One ivermectin-treated participant experienced a grade 3 ocular AE not considered a Mazzotti reaction: increased lacrimation starting 10 days after treatment. The logistic model showed a higher risk of at least one ocular AE within 6 months of treatment for women than men (*p* = 0.015, OR 1.404 with 95% CI 1.068–1.845) and a significant impact of mfAC levels at Month 1 (*p* = 0.0005 across all mfAC levels evaluated, OR 0 mfAC: > 10 mfAC 2.918 with 95% CI 1.737–4.9 and *p* < 0.0001). The OR for moxidectin:ivermectin was 1:0.709 (95% CI 0.536–0.939, *p* = 0.0165). Additional file 1: Table S9 shows the output of the final model.

## Discussion

In our study population from CDTI-naïve areas, selected to have at least 10 mf/mg skin, around 60% of participants had undetectable mfAC levels pre-treatment. Approximately 19% and 6% had mfAC levels between 1–5 and 6–10, respectively, and around 2% had > 40 mfAC across both eyes (Table 1). Pre-treatment SmfD significantly impacted pre-treatment mfAC levels (Table 7). Given that pre-treatment mfAC levels of > 10 mfAC increased the risk for at least one ocular Mazzotti reaction (Additional file 1, Table S6), it is noteworthy that this statistical significance occurs on the background of inter-individual variability in SmfD vs. mfAC levels (Additional file 1: Figs. S1 and S2). The levels of mfAC at a particular post-treatment timepoint were statistically significantly impacted by SmfD at that and/or previous timepoints as well as by mfAC levels at one or more previous timepoints (Table 7) but not by the treatment arm (Additional file 1: Table S4). We do not know what drives mfAC levels relative to SmfD levels to explain why SmfD are significantly lower at Months 1, 6, 12 and 18 after moxidectin than ivermectin treatment [21, 22, 30], but there is no treatment difference for mfAC levels.

Higher mfAC levels on Day 4 and Month 1 than pre-treatment were identified in 21% and 16% of moxidectin-treated and 19% and 15% of ivermectin-treated participants, respectively. This increase was not restricted to individuals with low mfAC levels pre-treatment (Tables 2, 5). A transient increase in mfAC levels in some individuals early after ivermectin as well as after DEC treatment has been shown previously, including during the clinical studies that supported regulatory approval of ivermectin [76–87]. The peak mfAC increase around Day 4 after treatment determined in these studies informed mfAC measurement on Day 3 or 4 in the moxidectin Phase 2 and 3 studies [21, 30]. An mfAC increase was not mentioned and cannot be deduced from the data provided in other publications of the early ivermectin studies [88–90]. Additional file 1: Table S10 provides an overview of the data reported. The way mfAC data are quantified in the publications (frequency of detectable mfAC levels, geometric means, descriptive text) makes it impossible to determine the frequency and extent of the mfAC increases for comparison with our data.

The lack of evaluation of mfAC changes early after treatment in subsequent studies of the efficacy and safety of ivermectin may be due to the fact that once the concern that ivermectin may have a similar risk of severe ocular reactions as DEC had been alleviated, quantifying changes in the number of ocular microfilariae early after ivermectin treatment was not considered important anymore. In the context of the proposed introduction of DEC as part of a 'pretreat and treat’ strategy into onchocerciasis elimination strategies [49], attention to mfAC levels before and increases early after treatment with DEC will become important in studies of the safety of the 'pretreat and treat’ strategy until a better understanding of the relationship between ocular mfAC before and early after treatment and the ocular safety of treatment with DEC has been achieved. In our study, pre-treatment mfAC levels > 10 significantly increased the risk of having at least one ocular Mazzotti reaction [occurring primarily within the first few days after treatment (Table 10)] and mfAC levels > 10 at Month 1 significantly increased the risk of at least one ocular AE within the first 6 months after treatment relative to 0 mfAC at these timepoints (Additional file 1: Tables S6, S9). In light of the fact that in our study women had a higher risk of having at least one ocular Mazzotti reaction and at least one ocular AE of any type than men (Additional file 1: Tables S6, S9), sex disaggregated analyses of the safety data of further studies of the ocular safety of ivermectin, DEC or moxidectin are indicated.

Within the context of the proposed introduction of DEC into onchocerciasis elimination strategies [49], a study of the tolerability and effect of ivermectin on skin and ocular microfilariae was recently conducted in primarily ivermectin-naïve individuals from hypoendemic villages in the Nkwanta North district of Ghana (pre-treatment SmfD 3 to 86.3 mf/mg). The study included mfAC measurement pre-treatment (64/231 with mfAC, mfAC range 1–150) and on Day 7, Month 3 and Month 6 post-treatment. The study identified a higher number of individuals with mfAC on Day 7 (73/231, mfAC range 1–82). The presentation of the data (frequency of detectable mfAC levels, geometric means, ranges and graphical presentation of individual participant data excluding the Day 7 data) [69] limits comparison with our data but the findings presented are consistent with our data and those of the early ivermectin studies. The lack of more detailed presentation of the mfAC data by Opoku et al. may be due to the fact that they attributed the mfAC increases to insensitivity of a single slit-lamp examination for detecting mfAC when counts are low, day to day variation in mfAC and easier detection of mfAC after they have been paralyzed by ivermectin [69]. The motility of mfAC during the first days after ivermectin treatment was found to be abnormal (transient immobility for 0.5–1 s, movement in extended rather than coiled configuration, motion in which mfAC bent only in the middle suggesting spasm) [88, 91] but paralysis facilitating counting has to our knowledge not been described previously. We observed in our study that mfAC were more mobile and difficult to count 3–4 days after treatment than at any other time in the study, resulting in frequent requests to participants to take a head-down position again for at least 5 min before counting of the mfAC in the second eye.

While we do not consider the increase in mfAC we observed early after treatment in around 20% of participants as due to methodological issues, we do recognize the limitations of the accuracy of mfAC counts. It is thus important not to overinterpret ‘0 mf counted’ as indicating that there are no mfAC in the eyes or a small change in mfAC counted as representing a real increase or decrease. However, we do think that the data available support the hypothesis that a ‘mobilization of microfilariae’ into the anterior chamber can occur. This term was used in some of the publications of the early ivermectin studies (e.g. [77, 78]). Mobilisation of microfilariae after DEC and ivermectin treatment into the urine and blood has been described previously as has appearance of microfilariae in other body fluids after DEC treatment [77, 78, 92–96]. We also observed mobilisation of microfilariae into blood and urine in some participants in the moxidectin Phase 2 study (unpublished data, summary statistics provided in Additional file 1: Table S11).

At Month 6 and 12, no mfAC were detected, respectively, in 95% and 98% of participants who had received a single dose of moxidectin and in 95% and 96% of participants who had received a single dose of ivermectin. At these timepoints, > 10 mfAC were detected in 0.2% and 0.2% of moxidectin- and in 0.4% and 0.2% of ivermectin-treated participants, respectively (Tables 3, 4). The above referenced study by Opoku and co-workers [69] identified mfAC 6 months after ivermectin treatment in a comparable percentage of participants [12/212 (5.7%), mfAC range 1–35], including ‘low mfAC counts’ in three participants who had no mfAC detected pre-treatment (but two had mfAC detected on Day 7). In our study, covariates impacting Month 6 mfAC levels significantly were Month 1 SmfD and Day 4 and Month 1 mfAC levels. Covariates with significant impact on Month 12 mfAC levels were SmfD pre-treatment and at Month 12 as well as mfAC levels at pre-treatment, Month 1 and 6 (Table 7). Repeat dose studies will allow us to determine whether the covariates which impacted mfAC levels in our study are also impacting mfAC levels after multiple treatments. Data analysis not only for inter-individual but also intra-individual variability in changes in SmfD and mfAC levels is needed to determine whether individuals show the same pattern of post-treatment changes in SmfD and mfAC levels after each treatment or not. Depending on the outcome of large-scale studies of the safety of the ‘pretreat and treat’ strategy, a non-random response pattern will have implications for assessing the risk of this strategy. As recently pointed out [97], a non-random response pattern also has implications for the prospects of achieving onchocerciasis elimination with ivermectin (conferring an additional advantage to moxidectin-based strategies). This will affect the comparative benefit-risk assessment of MDAi vs. different ATS required for a decision on whether or not to include a ‘pre-treat and treat’ strategy in onchocerciasis elimination guidelines and policies.

## Conclusions

Among our ivermectin-naïve study participants from onchocerciasis meso- or hyperendemic villages with at least 10 mf/mg skin, there was no statistically significant difference in the effect of a single dose of 8 mg moxidectin or 150 µg/kg ivermectin on mfAC levels on Day 4 or Month 1, 6, 12 or 18. Ocular Mazzotti reactions occurred in 12.4% of moxidectin and 10.2% of ivermectin treated participants without a difference in the type of these reactions or their severity between the treatment arms. The extent to which findings on the efficacy and safety of ivermectin, moxidectin and DEC in ivermectin-naïve individuals are relevant for decisions on including DEC into strategies for elimination of onchocerciasis in areas with a long history of MDAi needs to be determined. Assuming the benefit of a ‘pre-treat (with ivermectin) and treat (with IDA)’ strategy for elimination of *O. volvulus* transmission compared to strategies without DEC is demonstrated, additional studies are required to support an assessment of the risks of this strategy. Our results, as well as the results of the early ivermectin studies and past experience with DEC treatment (Additional file 1: Table S10), suggest that safety studies should be designed to improve understanding of (i) the role of SmfD and mfAC levels pre-treatment and of mobilisation of microfilariae into the anterior chambers early after treatment on the safety of DEC and (ii) inter-individual susceptibility to adverse reactions to DEC treatment, ideally including the determinants. Safety study design should also consider the percentage of the population which participates irregularly in MDAi (see e.g. [60, 61, 67, 98, 99]) and the effect of irregular MDAi participation on SmfD as well as the potential for higher risk of women than men for post-treatment AEs. Decisions on the risk-benefit ratio of strategies including DEC should interindividual variability in skin and/or ocular microfilariae levels, inter- and intra-individual variability in response to treatment, interindividual variability in frequency, type and severity of adverse reactions as well as possible differences between onchocerciasis-endemic areas [22]. The decisions should carefully weigh any projected population-based benefits vs. the risks to even a small percentage of individuals. If the benefit of the proposed ‘pretreat and treat’ strategy compared to MDAi or to other ATS is demonstrated, knowledge of the determinants of adverse reactions to DEC treatment in even a very small percentage of individuals could pave the way to ATS which exclude individuals at risk from DEC treatment, as was done for the small percentage of individuals with high *Loa loa* microfilaraemia at risk of severe and serious reactions to ivermectin [31, 42–46, 100, 101].

## Copyright

World Health Organization 2023. This article is published under the CC BY 3.0 IGO license (https://creativecommons.org/licenses/by/3.0/igo) which permits use, sharing, adaptation, distribution, and reproduction in any medium or format, as long as the original work is properly cited, appropriate credit is given to the IGO, a link is provided to the Creative Commons licence, and any changes made are indicated. In any use of this article, there should be no suggestion that WHO endorses any specific organization, products, or services. The use of the WHO logo is not permitted. This notice should be preserved along with the article’s original URL.

### Supplementary Information

**Additional file 1: Fig S1.** mfAC pre-treatment, 4 days, 1, 6, 12 and 18 months post-treatment among participants with < 10 mfAC pre-treatment by pre-treatment SmfD. **Fig S2.** mfAC pre-treatment, 4 days, 1, 6, 12 and 18 months post-treatment among participants with ≥ 10 mfAC pre-treatment by pre-treatment SmfD. **Fig S3.** mfAC 1, 6, 12 and 18 months post-treatment among participants with < 10 mfAC pre-treatment by post-treatment SmfD. **Fig S4.** mfAC 1, 6, 12 and 18 months post-treatment among participants with ≥ 10 mfAC pre-treatment by post-treatment SmfD. **Table S1.** Participants with both eyes evaluated at each timepoint by pre-treatment SmfD and mfAC category and sex. **Table S2.** Mean (SD), minimum and maximum mfAC levels among participants with increases from pre-treatment to Day 4 or Month 1 and from Day 4 to Month 1 resulting in mfAC levels in a higher mfAC category. **Table S3.** Number (%) of participants by mfAC category on Day 4 or Month 1 after treatment by pre-treatment and Day 4 mfAC category and treatment arm. **Table S4.** Output linear model for mfAC. **Table S5.** Ocular medical history of study participants by study area. **Table S6.** Output of final logistic model of the factors impacting the risk to have at least one ocular Mazzotti reaction. **Table S7.** Number (%) of participants with ocular adverse events starting within 1 and between month 1 and end of 6 months after treatment by ocular AE based on MedDRA coding. **Table S8.** Number of ocular adverse events starting within 1 and between month 1 and end of 6 months after treatment by ocular AE based on MedDRA coding. **Table S9.** Output of final logistic model of the factors and covariates impacting the risk to have at least one ocular adverse event. **Table S10.** Literature data on mfAC number change early after treatment with diethylcarbamazine, ivermectin and suramin. **Table S11.** Microfilariae in the blood and urine before and after treatment (data from the moxidectin Phase 2 study, across all participants and for participants with > 20mf/mg skin).

## Data Availability

Participants consented to publication of summaries of the results, not to sharing of their individual data. Consequently, the Sponsor (WHO) and the authors do not have the participants’ permission to make individual participant data publicly available. Individuals wanting to analyze the data should contact the Sponsor (tdr@who.int) and Medicines Development for Global Health to which WHO has licensed the data (via mark. sullivant@medicinesdevelopment.com or https:// www.medicinesdevelopment.com/contact). Requests should include the objectives, data analysis plan and plans to obtain applicable Ethics Committee approvals and involve the investigators (co-authors on this manuscript) and commitment to not share the data with anybody else.

## Abbreviations

AE: Adverse event
CDTI: Community directed treatment with ivermectin
CI: 95% Confidence interval
Day 4: Day 4 after treatment
DEC: Diethylcarbamazine
IA: Ivermectin, albendazole
IDA: Ivermectin, diethylcarbamazine and albendazole
IoI: Intensity of infection (pre-treatment skin microfilariae density)
Month 1, 6, 12, 18: Month 1, 6, 12, 18 after treatment
MDAi: Mass drug administration of ivermectin
MedDRA: Medical Dictionary for Regulatory Activities
mf: Microfilaria
mfAC: Live microfilariae in the anterior chambers
mfCOR: Microfilariae in the cornea
OCRC: Onchocerciasis Chemotherapy Research Center (Hohoe, Ghana)
OR: Odds ratio
PreTx: Pre-treatment
SmfD: Skin microfilariae density (mf/mg skin)
