# Supplementary material for: Onchocerca volvulus microfilariae in the anterior chambers of the eye and ocular adverse events after a single dose of 8 mg moxidectin or 150 µg/kg ivermectin: results of a randomized double-blind Phase 3 trial in the Democratic Republic of the Congo, Ghana and Liberia
Source: Parasit Vectors. 2024 Mar 15;17:137. doi: 10.1186/s13071-023-06087-3 (PMC10943894; doi:10.1186/s13071-023-06087-3)
Supplement: Supplementary file 1 — Additional file 1: Fig S1. mfAC pre-treatment, 4 days, 1, 6, 12 and 18 months post-treatment among participants with < 10 mfAC pre-treatment by pre-treatment SmfD. Fig S2. mfAC pre-treatment, 4 days, 1, 6, 12 and 18 months post-treatment among participants with ≥ 10 mfAC pre-treatment by pre-treatment SmfD. Fig S3. mfAC 1, 6, 12 and 18 months post-treatment among participants with < 10 mfAC pre-treatment by post-treatment SmfD. Fig S4. mfAC 1, 6, 12 and 18 months post-treatment among participants with ≥ 10 mfAC pre-treatment by post-treatment SmfD. Table S1. Participants with both eyes evaluated at each timepoint by pre-treatment SmfD and mfAC category and sex. Table S2. Mean (SD), minimum and maximum mfAC levels among participants with increases from pre-treatment to Day 4 or Month 1 and from Day 4 to Month 1 resulting in mfAC levels in a higher mfAC category. Table S3. Number (%) of participants by mfAC category on Day 4 or Month 1 after treatment by pre-treatment and Day 4 mfAC category and treatment arm. Table S4. Output linear model for mfAC. Table S5. Ocular medical history of study participants by study area. Table S6. Output of final logistic model of the factors impacting the risk to have at least one ocular Mazzotti reaction. Table S7. Number (%) of participants with ocular adverse events starting within 1 and between month 1 and end of 6 months after treatment by ocular AE based on MedDRA coding. Table S8. Number of ocular adverse events starting within 1 and between month 1 and end of 6 months after treatment by ocular AE based on MedDRA coding. Table S9. Output of final logistic model of the factors and covariates impacting the risk to have at least one ocular adverse event. Table S10. Literature data on mfAC number change early after treatment with diethylcarbamazine, ivermectin and suramin. Table S11. Microfilariae in the blood and urine before and after treatment (data from the moxidectin Phase 2 study, across all participants and for participants wi [file 13071_2023_6087_MOESM1_ESM.pdf]

Additional File 1

Onchocerca volvulus microfilariae in the anterior chambers of the eye and ocular adverse events after a single dose of 8 mg moxidectin or 150 µg/kg ivermectin: Results of a randomized double-blind Phase 3 trial in the Democratic Republic of the Congo, Ghana and Liberia

Short title: Ocular *O. volvulus* microfilariae after moxidectin or ivermectin

Eric M Kanza[1,#a*], Amos Nyathirombo[2,#b*], Jemmah P. Larbelee[3,#c*], Nicholas O Opoku[4,#d], Didier K. Bakajika[2,#e], Hayford M Howard[3,#f], Germain L Mambandu[2,#g], Maurice M Nigo[2,#h], Deogratias Ucima Wonyarossi[2], Françoise Ngave[2], Kambale Kasonia Kennedy[1,#i], Kambale Kataliko[2,#j], Kpehe M Bolay[3,#k], Simon K Attah[4,#l], George Olipoh[4,#m], Sampson Asare[4,#n], Mupenzi Mumbere[1#o], Michel Vaillant[5], Christine M Halleux[6], Annette C Kuesel[6#p**]

[1] Centre de Recherche Clinique de Butembo, Université Catholique du Graben, Site Horizon, Butembo, Nord Kivu, DRC
[2] Centre de Recherche en Maladies Tropicale de l'Ituri, Hôpital Générale de Référence de Rethy, Ituri, Democratic Republic of the Congo (DRC)
[3] Clinical Research Center, Liberia Institute for Biomedical Research, Bolahun, Liberia
[4] Onchocerciasis Chemotherapy Research Center, Hohoe, Ghana
[5] Competence Center for Methodology and Statistics, Luxembourg Institute of Health, Strassen, Grand Duchy of Luxembourg
[6] UNICEF/UNDP/World Bank/WHO Special Programme for Research and Training in Tropical Diseases (WHO/TDR), World Health Organization, Geneva, Switzerland

[#a] Current Address: Programme National de Lutte contre les Maladies Tropicales Négligées à Chimio-Thérapie Préventive (PNLMTN-CTP), Democratic Republic of the Congo
[#b] Current Address: Department of Ophthalmology, Faculty of Medicine, Gulu University, Uganda
[#c] Current Address: Hospital, Monrovia, Liberia
[#d] Current Address: Department of Epidemiology and Biostatistics School of Public Health, University of Health and Allied Sciences, Hohoe, Ghana
[#e] Current Address: ESPEN, African Regional Office of the World Health Organization (WHO/AFRO/ESPEN), Brazzaville, Republic of Congo
[#f] Current Address: Ganta United Methodist Hospital, Ganta City, Nimba County, Liberia
[#g] Current Address: Inspection Provinciale de la Santé de la Tshopo, Division Provinciale de la Santé de la Tshopo, Kisangani, Province de la Tshopo, DRC
[#h] Current Address: Institut Supérieur des Techniques Médicales de Nyankunde, Bunia, Ituri, DRC
[#i] Current Address: Department of Clinical Research, London School of Hygiene and Tropical Medicine, UK
[#j] Current Address: Centre de Santé CECA 20 de Mabakanga, Beni, Nord Kivu, DRC.
[#k] Current Address: National Public Health Institute of Liberia, Public Health & Medical Research, Monrovia, Liberia
[#l] Current Address: Department of Microbiology, University of Ghana Medical School, Accra, Ghana, Baldwin University College, Accra
[#m] Current Address: Precious Minerals Marketing Company Ltd., National Assay Centre, Technical Department, Diamond House, Accra, Ghana
[#n] Current Address: Bell Laboratories Inc, Window, Wisconsin, United States of America
[#o] Current Address: Medicines Development for Global Health (MGDH), Melbourne, Australia
[#p] Current Address: retired since 1 March 2023
* Joint first authors

**Corresponding author: kuesela@who.int , annette.kuesel@mailbox.org ORCID ID: 0000-0002-1696-1784

Table of Contents

Fig S1 mfAC pre-treatment, 4 days, 1, 6, 12 and 18 months post-treatment among participants with <10 mfAC pre-treatment by pre-treatment SmfD 3
Fig S2 mfAC pre-treatment, 4 days, 1, 6, 12 and 18 months post-treatment among participants with ≥10 mfAC pre-treatment by pre-treatment SmfD 4
Fig S3 mfAC 1, 6, 12 and 18 months post-treatment among participants with <10 mfAC pre-treatment by post-treatment SmfD 5
Fig S4 mfAC 1, 6, 12 and 18 months post-treatment among participants with ≥10 mfAC pre-treatment by post-treatment SmfD 6
Table S1: Participants with both eyes evaluated at each time point by pre-treatment SmfD and mfAC category and sex 7
Table S2: Mean (SD), minimum and maximum mfAC levels among participants with increases from pre-treatment to Day 4 or Month 1 and from Day 4 to Month 1 resulting in mfAC levels in a higher mfAC category 8
Table S3: Number (%) of participants by mfAC category on Day 4 or Month 1 after treatment by pre-treatment and Day 4 mfAC category and treatment arm 10
Table S4: Output linear model for mfAC 11
Table S5: Ocular medical history* of study participants by study area 13
Table S6: Output of final logistic model of the factors impacting the risk to have at least one ocular Mazzotti reaction 16
Table S7: Number (%) of participants with ocular adverse events starting within 1 and between month 1 and end of 6 months after treatment by ocular AE based on MedDRA coding 17
Table S8: Number of ocular adverse events starting within 1 and between month 1 and end of 6 months after treatment by ocular AE based on MedDRA coding 19
Table S9: Output of final logistic model of the factors and covariates impacting the risk to have at least one ocular adverse event 21
Table S10: Literature data on mfAC number change early after treatment with diethylcarbamazine, ivermectin and suramin 22
Table S11: Microfilariae in the blood and urine before and after treatment (data from the moxidectin Phase 2 study, across all participants and for participants with > 20mf/mg skin) 26
REFERENCES 28

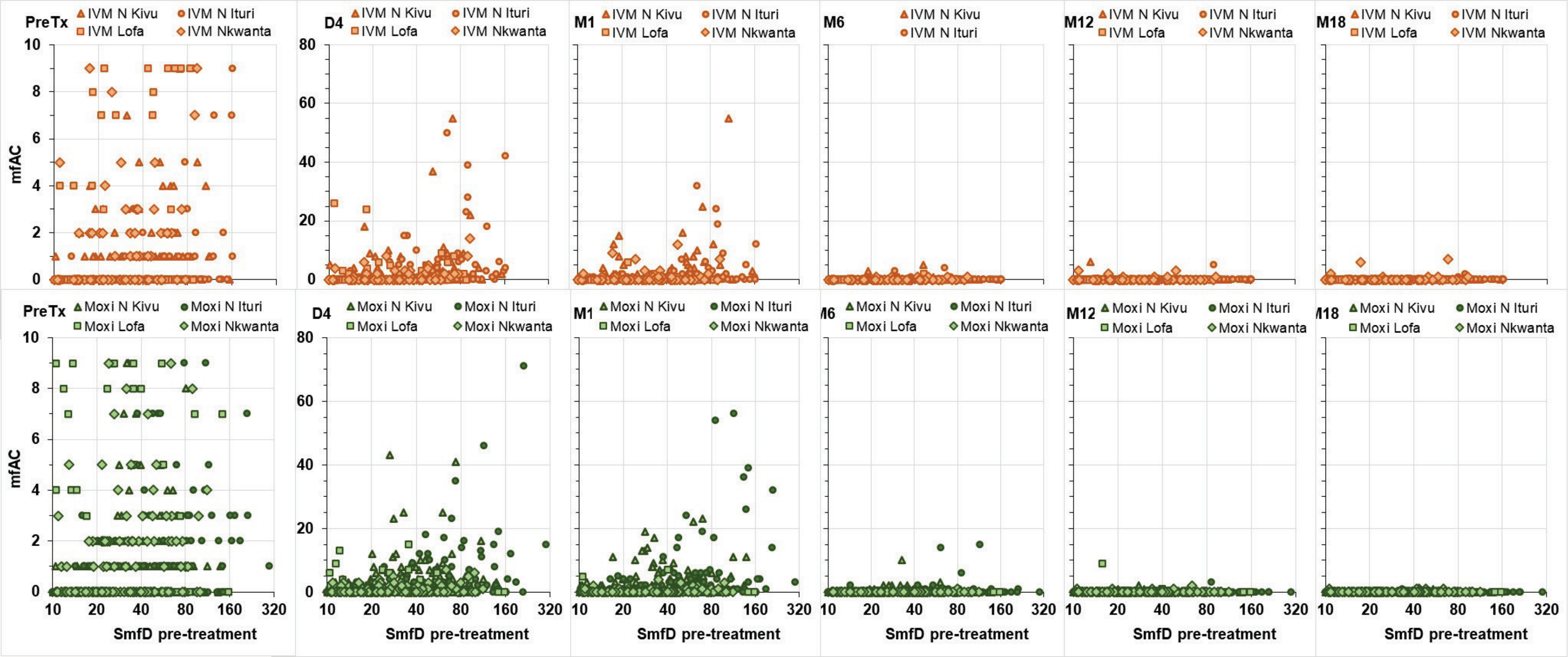

Fig S1 **mfAC pre-treatment, 4 days, 1, 6, 12 and 18 months post-treatment among participants with <10 mfAC pre-treatment by pre-treatment SmfD**

mfAC microfilariae in the anterior chambers, PreTx before treatment, D4 4 days after treatment, M1 M6, M12, M18, 1, 6, 12 and 18 months after treatment. x-axis pre-treatment SmfD, y-axis mfAC levels preTx, D4, M1, M6, M12, M18 (in column 1, 2, 3, 4, 5, 6, respectively) post-treatment with ivermectin (IVM, upper row, orange symbols) or moxidectin (Moxi, lower row, green symbols). Data from participants from Nord Kivu (∆), Nord Ituri (○), Lofa County (□) and Nkwanta district (◊).

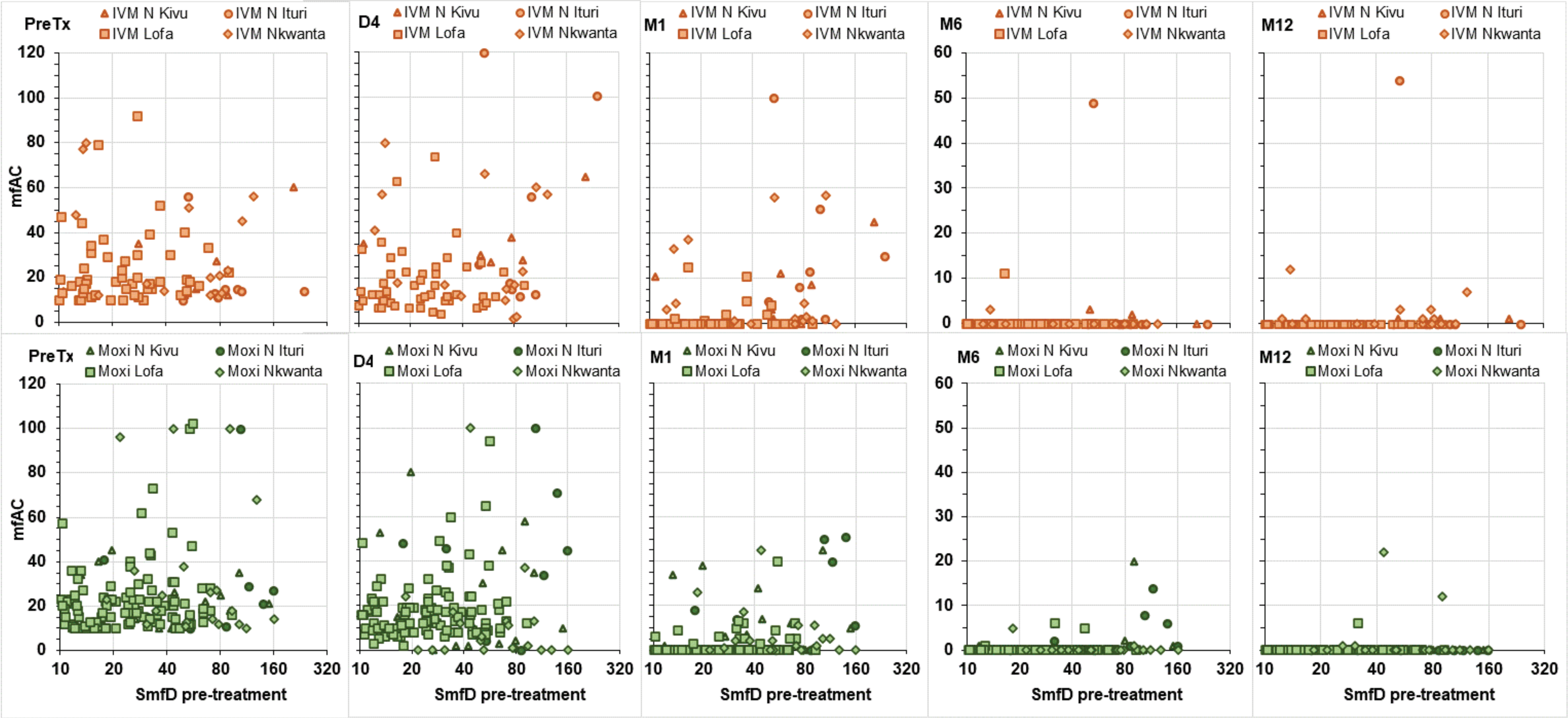

Fig S2 **mfAC pre-treatment, 4 days, 1, 6, 12 and 18 months post-treatment among participants with ≥10 mfAC pre-treatment by pre-treatment SmfD**

mfAC microfilariae in the anterior chambers, PreTx before treatment, D4 4 days after treatment, M1 M6, M12, M18, 1, 6, 12 and 18 months after treatment. x-axis pre-treatment SmfD, y-axis mfAC levels preTx, D4, M1, M6, M12, M18 (in column 1, 2, 3, 4, 5, 6, respectively) post-treatment with ivermectin (IVM, upper row, orange symbols) or moxidectin (Moxi, lower row, green symbols). Data from participants from Nord Kivu (∆), Nord Ituri (○), Lofa County (□) and Nkwanta district (◊).

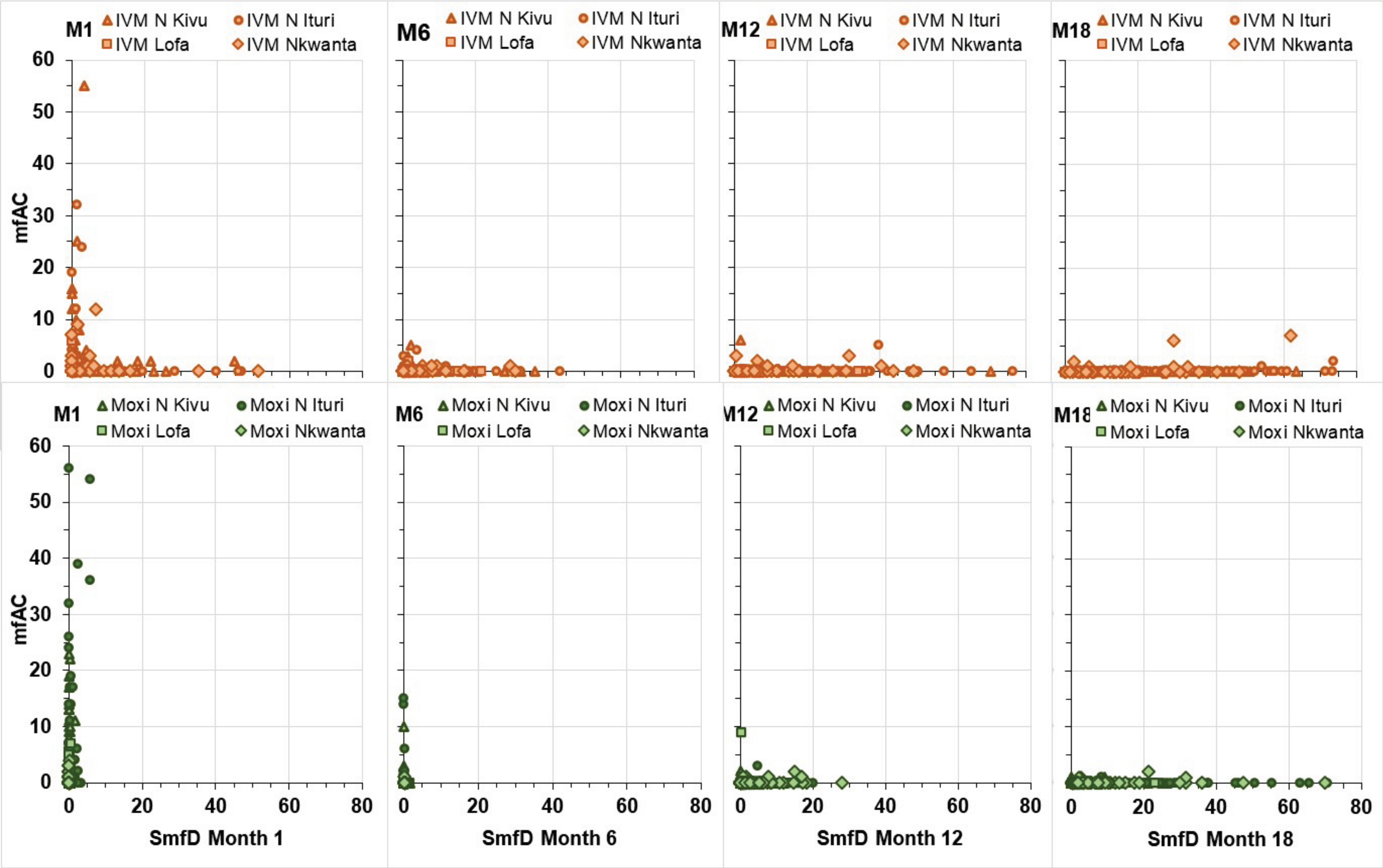

Fig S3 mfAC 1, 6, 12 and 18 months post-treatment among participants with <10 mfAC pre-treatment by post-treatment SmfD

mfAC microfilariae in the anterior chambers, D4, 4 days after treatment, M1 M6, M12, M18, 1, 6, 12 and 18 months after treatment.
x-axis SmfD, y axis mfAC levels at M1, M6, M12, M18 (in column 1, 2, 3, 4, respectively) post-treatment with ivermectin (IVM, upper row, orange symbols) or moxidectin (Moxi, lower row, green symbols). Data from participants from Nord Kivu (∆), Nord Ituri (○), Lofa County (□) and Nkwanta district (◊).

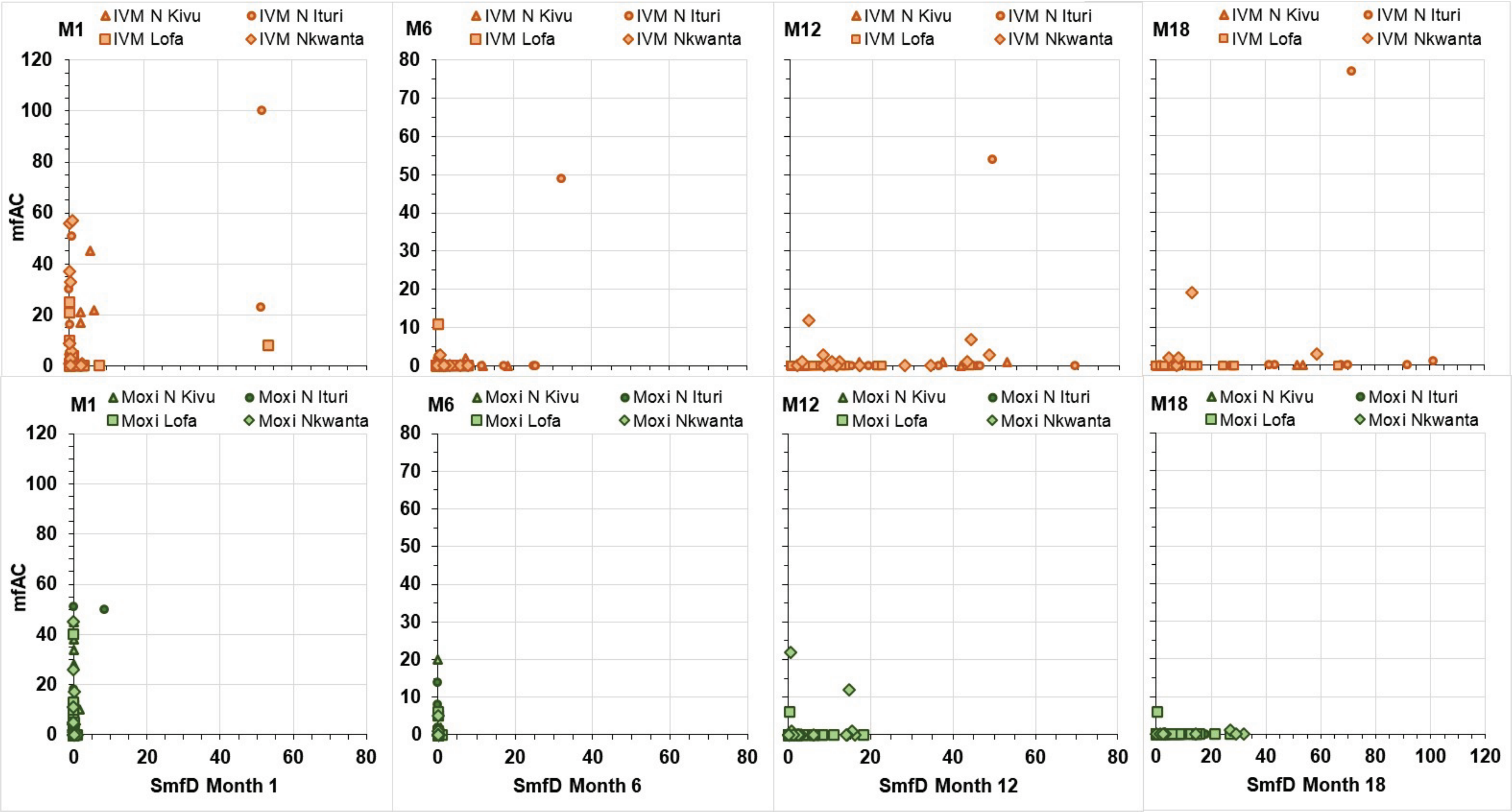

Fig S4 mfAC 1, 6, 12 and 18 months post-treatment among participants with ≥10 mfAC pre-treatment by post-treatment SmfD

mfAC microfilariae in the anterior chambers, D4, 4 days after treatment, M1 M6, M12, M18, 1, 6, 12 and 18 months after treatment.

x-axis SmfD, y axis mfAC levels at M1, M6, M12, M18 (in column 1, 2, 3, 4, respectively) post-treatment with ivermectin (IVM, upper row, orange symbols) or moxidectin (Moxi, lower row, green symbols). Data from participants from Nord Kivu (Δ), Nord Ituri (○), Lofa County (□) and Nkwanta district (◊).

Table S1: Participants with both eyes evaluated at each time point by pre-treatment SmfD and mfAC category and sex

PreTx SmfD	PreTx mfAC	Women		Men		Any sex	
		n	%[1]	n	%[1]	n	%[2]
<20	0	113	38	187	62	300	70.3
	1-5	20	47	23	53	43	10.1
	6-10	9	47	10	53	19	4.4
	11-20	12	36	21	64	33	7.7
	21-40	11	46	13	54	24	5.6
	>40	5	63	3	38	8	1.9
	Any	170	40	257	60	427	100.0
20-<50	0	126	31	276	69	402	63.3
	1-5	46	38	74	62	120	18.9
	6-10	17	46	20	54	37	5.8
	11-20	14	35	26	65	40	6.3
	21-40	9	35	17	65	26	4.1
	>40	4	40	6	60	10	1.6
	Any	216	34	419	66	635	100.0
≥50	0	51	28	132	72	183	45.6
	1-5	46	40	70	60	116	28.9
	6-10	18	56	14	44	32	8.0
	11-20	16	41	23	59	39	9.7
	21-40	6	30	14	70	20	5.0
	>40	4	36	7	64	11	2.7
	Any	141	35	260	65	401	100.0

[1] percentage calculated by pre-treatment SmfD and mfAC category, [2] percentage calculated by mfAC category within the SmfD category

Table S2: Mean (SD), minimum and maximum mfAC levels among participants with increases from pre-treatment to Day 4 or Month 1 and from Day 4 to Month 1 resulting in mfAC levels in a higher mfAC category

					1-5 mfAC				6-10 mfAC				11-20 mfAC				21-40 mfAC				>40 mfAC			
mfAC	AM	SD	Min	Max	AM	SD	Min	Max	AM	SD	Min	Max	AM	SD	Min	Max	AM	SD	Min	Max	AM	SD	Min	Max
	Moxidectin																							
	PreTx				Day 4																			
0	0	0	0	0	1.60	0.96	1	5	7.09	1.04	6	9	15.67	2.52	13	18	23.00	0	23	23				
1-5	2.39	1.50	1	5					7.88	1.54	6	10	13.85	2.41	11	19	27.67	6.43	23	35	50.25	13.99	41	71
6-10	7.67	1.50	6	10									12.67	2.08	11	15	26.50	6.45	22	36	51.00	5.66	47	55
11-20	15.00	3.00	12	18													30.00	0	30	30	52.00	8.49	46	58
21-40	26.00	5.94	21	34																	53.50	12.26	45	71
All	1.50	4.39	0	34	1.60	0.96	1	5	7.56	1.4	6	10	13.95	2.41	11	19	26.89	5.42	22	36	51.75	10.29	41	71
	PreTx				Month 1																			
0	0	0	0	0	1.87	1.18	1	5	7.40	1.34	6	9	16.50	3.54	14	19	26.00	0	26	26				
1-5	2.40	1.53	1	5					6.78	1.30	6	10	13.33	2.92	11	19	30.40	7.64	22	39	55.00	1.41	54	56
6-10	7.25	1.49	6	10									14.75	2.87	11	17	25.67	2.08	24	28	75.00	0	75	75
11-20	12.00		12	12													40	0	40	40				
21-40	28.00	9.90	21	35																	48.00	4.24	45	51
All	1.18	3.78	0	35	1.87	1.18	1	5	7	1.30	6	10	14.13	2.97	11	19	29.5	6.77	22	40	56.2	11.3	45	75
	Day 4				Month 1																			
0	0	0	0	0	1.81	1.18	1	5	6.78	0.97	6	9	12.50	2.12	11	14								
1-5	2.50	1.17	1	5					7.50	1.29	6	9	11.00	0	11	11	25.67	2.52	23	28	54	0	54	54
6-10	8.40	0.89	7	9									12.50	0.71	12	13	28.67	9.87	22	40				
11-20	17.00	2.83	15	19													37.50	2.12	36	39				
21-40	35.50	0.71	35	36																	60	21.21	45	75
All	1.77	5.72	0	36	1.81	1.18	1	5	7	1.08	6	9	11.75	1.16	11	14	29.75	7.42	22	40	58	15.39	45	75
	Ivermectin																							
	PreTx				Day 4																			
0	0	0	0	0	1.71	1.12	1	5	6.50	0.58	6	7	11.00		11	11	24.50	2.12	23	26				
1-5	2.45	1.37	1	5					8.00	1.36	6	10	16.00	1.73	15	18	27.75	6.65	22	37				
6-10	8.86	0.90	7	10									16.00	2.83	14	18	32.50	9.19	26	39	49.00	6.56	42	55
11-20	13.29	1.60	11	15													28.40	4.72	22	35	78.50	31.82	56	101
21-40																								
All	2.27	4.06	0	15	1.71	1.12	1	5	7.68	1.38	6	10	15.17	2.64	11	18	28.23	5.6	22	39	60.80	23.15	42	101
	PreTx				Month 1																			

					1-5 mfAC				6-10 mfAC				11-20 mfAC				21-40 mfAC				>40 mfAC			
**mfAC**	**AM**	**SD**	**Min**	**Max**	**AM**	**SD**	**Min**	**Max**	**AM**	**SD**	**Min**	**Max**	**AM**	**SD**	**Min**	**Max**	**AM**	**SD**	**Min**	**Max**	**AM**	**SD**	**Min**	**Max**
0	0	0	0	0	1.76	0.91	1	4	7.33	1.21	6	9					24.00		24	24				
1-5	2.80	1.62	1	5					8.00	2.00	6	10	14.33	2.88	12	19					55.00	0	55	55
6-10	9.00	0.00	9	9									12.00		12	12	28.50	4.95	25	32				
11-20	14.17	1.17	12	15													26.60	6.80	21	37	51.00	0	51	51
21-40																								
All	1.97	4.28	0	15	1.76	0.91	1	4	7.56	1.42	6	10	14	2.77	12	19	26.75	5.65	21	37	53.00	2.83	51	55
	**Day 4**				**Month 1**																			
0	0	0	0	0	1.59	0.86	1	4	7.25	1.5	6	9												
1-5	2.80	1.79	1	5					7.00	1.00	6	8	12.00	0	12	12					55.00	0	55	55
6-10	9.00	0	9	9									13.5	2.12	12	15								
11-20	15.00	4.24	12	18													30.00	9.90	23	37				
21-40																								
All	1.24	3.51	0	18	1.59	0.86	1	4	7.14	1.21	6	9	13.00	1.73	12	15	30.00	9.90	23	37	55.00	0	55	55

AM Arithmetic Mean, SD Standard Deviation, Max maximum, mfAC live microfilariae in the anterior chambers, Min minimum, PreTx pre-treatment

Table S3: Number (%) of participants by mfAC category on Day 4 or Month 1 after treatment by pre-treatment and Day 4 mfAC category and treatment arm

	Moxidectin													Ivermectin												
		0 mfAC		1-5 mfAC		6-10 mfAC		11-20 mfAC		21-40 mfAC		>40 mfAC			0 mfAC		1-5 mfAC		6-10 mfAC		11-20 mfAC		21-40 mfAC		>40 mfAC	
mfAC	N	n	%	n	%	n	%	n	%	n	%	n	%	N	n	%	n	%	n	%	n	%	n	%	n	%
**PreTx**	**Day 4**													**Day 4**												
0	596	446	74.8	135	22.7	11	1.8	3	0.5	1	0.2			289	233	80.6	49	17.0	4	1.4	1	0.3	2	0.7		
1-5	183	65	35.5	82	44.8	16	8.7	13	7.1	3	1.6	4	2.2	96	31	32.3	43	44.8	15	15.6	3	3.1	4	4.2		
6-10	59	15	25.4	16	27.1	19	32.2	3	5.1	4	6.8	2	3.4	29	1	3.4	9	31.0	12	41.4	2	6.9	2	6.9	3	10.3
11-20	67	5	7.5	3	4.5	25	37.3	31	46.3	1	1.5	2	3.0	45			3	6.7	11	24.4	24	53.3	5	11.1	2	4.4
21-40	52	1	1.9	4	7.7	1	1.9	23	44.2	19	36.5	4	7.7	18			1	5.6			4	22.2	13	72.2		
>40	16	2	12.5		0.0		0.0		0.0	4	25.0	10	62.5	13									3	23.1	10	76.9
All	973	534	54.9	240	24.7	72	7.4	73	7.5	32	3.3	22	2.3	490	265	54.1	105	21.4	42	8.6	34	6.9	29	5.9	15	3.1
**PreTx**	**Month 1**													**Month 1**												
0	592	471	79.6	113	19.1	5	0.8	2	0.3	1	0.2			287	235	81.9	45	15.7	6	2.1			1	0.3		
1-5	181	92	50.8	64	35.4	9	5.0	9	5.0	5	2.8	2	1.1	96	51	53.1	35	36.5	3	3.1	6	6.3			1	1.0
6-10	59	34	57.6	10	16.9	7	11.9	4	6.8	3	5.1	1	1.7	29	18	62.1	3	10.3	5	17.2	1	3.4	2	6.9		
11-20	67	48	71.6	7	10.4	5	7.5	6	9.0	1	1.5		0.0	45	24	53.3	11	24.4	2	4.4	2	4.4	5	11.1	1	2.2
21-40	52	37	71.2	6	11.5	1	1.9	3	5.8	3	5.8	2	3.8	18	15	83.3	1	5.6	2	11.1						
>40	16	9	56.3	2	12.5			2	12.5	1	6.3	2	12.5	13	4	30.8			2	15.4			3	23.1	4	30.8
All	967	691	71.5	202	20.9	27	2.8	26	2.7	14	1.4	7	0.7	488	347	71.1	95	19.5	20	4.1	9	1.8	11	2.3	6	1.2
**Day 4**	**Month 1**													**Month 1**												
0	533	454	85.2	68	12.8	9	1.7	2	0.4		0.0		0.0	263	222	84.4	37	14.1	4	1.5						
1-5	237	129	54.4	96	40.5	4	1.7	4	1.7	3	1.3	1	0.4	105	62	59.0	38	36.2	3	2.9	1	1.0			1	1.0
6-10	71	39	54.9	18	25.4	9	12.7	2	2.8	3	4.2		0.0	42	26	61.9	10	23.8	4	9.5	2	4.8				
11-20	72	45	62.5	12	16.7	4	5.6	9	12.5	2	2.8		0.0	34	20	58.8	6	17.6	4	11.8	2	5.9	2	5.9		
21-40	32	18	56.3	5	15.6	1	3.1	4	12.5	2	6.3	2	6.3	29	15	51.7	4	13.8	3	10.3	3	10.3	4	13.8		
>40	22	6	27.3	3	13.6		0.0	5	22.7	4	18.2	4	18.2	15	2	13.3			2	13.3	1	6.7	5	33.3	5	33.3
All	967	691	71.5	202	20.9	27	2.8	26	2.7	14	1.4	7	0.7	488	347	71.1	95	19.5	20	4.1	9	1.8	11	2.3	6	1.2

mfAC live microfilariae in the anterior chambers

Table S4: Output linear model for mfAC

Baseline mfAC		Type 3 Tests of Fixed Effects				
**Dependent Variable**	log_anterior_chamber_baseline	**Effect**	**Num DF**	**Den DF**	**F Value**	**p-value**
		**AGE**	72	1384	1.23	0.0973
		**SEX**	1	1384	2.81	0.094
		**txgroup**	1	1384	0.7	0.404
		**log_skin_density_baseline**	1	1384	90.21	<.0001
**D4 mfAC**		**Type 3 Tests of Fixed Effects**				
**Dependent Variable**	log_anterior_chamber_D4	**Effect**	**Num DF**	**Den DF**	**F Value**	**Pr > F**
		**AGE**	72	1383	1.16	0.1701
		**SEX**	1	1383	2.75	0.0974
		**txgroup**	1	1383	1.74	0.1873
		**log_anterior_chamber_baseline**	1	1383	1838.64	<.0001
		**log_skin_density_baseline**	1	1383	11.91	0.0006
**M1 mfAC**		**Type 3 Tests of Fixed Effects**				
**Dependent Variable**	log_Sum_anterior_chamber_M1	**Effect**	**Num DF**	**Den DF**	**F Value**	**Pr > F**
		**AGE**	72	1373	1.4	0.017
		**SEX**	1	1373	1.61	0.204
		**txgroup**	1	1373	2.68	0.1016
		**log_anterior_chamber_baseline**	1	1373	2.11	0.1469
		**log_skin_density_baseline**	1	1373	36.68	<.0001
		**log_anterior_chamber_D4**	1	1373	208.84	<.0001
		**log_skin_density_M1**	1	1373	7.63	0.0058
**M6 mfAC**		**Type 3 Tests of Fixed Effects**				
**Dependent Variable**	log_Sum_anterior_chamber_M6	**Effect**	**Num DF**	**Den DF**	**F Value**	**Pr > F**
		**AGE**	71	1353	0.81	0.868
		**SEX**	1	1353	1.75	0.1862
		**txgroup**	1	1353	0.45	0.5016
		**log_anterior_chamber_baseline**	1	1353	0.25	0.615
		**log_skin_density_baseline**	1	1353	0	0.9828
		**log_anterior_chamber_D4**	1	1353	4.15	0.0419
		**log_Sum_anterior_chamber_M1**	1	1353	112.53	<.0001
		**log_skin_density_M1**	1	1353	14.23	0.0002
		**log_skin_density_M6**	1	1353	2.01	0.1561
**M12 mfAC**		**Type 3 Tests of Fixed Effects**				

Baseline mfAC		Type 3 Tests of Fixed Effects				
**Dependent Variable**	log_Sum_anterior_chamber_M12	**Effect**	**Num DF**	**Den DF**	**F Value**	**p-value**
		**AGE**	71	1319	0.82	0.8605
		**SEX**	1	1319	0.08	0.7781
		**txgroup**	1	1319	1.53	0.2168
		**log_Sum_anterior_chamber_baseline**	1	1319	10.1	0.0015
		**log_skin_density_baseline**	1	1319	10.09	0.0015
		**log_anterior_chamber_D4**	1	1319	1.24	0.2651
		**log_Sum_anterior_chamber_M1**	1	1319	20.08	<.0001
		**log_skin_density_M1**	1	1319	3.71	0.0544
		**log_Sum_anterior_chamber_M6**	1	1319	57.67	<.0001
		**log_skin_density_M6**	1	1319	0.06	0.8066
		**log_skin_density_M12**	1	1319	10.86	0.001

M18 mfAC		Type 3 Tests of Fixed Effects				
**Dependent Variable**	log_Sum_anterior_chamber_M18	**Effect**	**Num DF**	**Den DF**	**F Value**	**p-value**
		**AGE**	71	1039	0.59	0.997
		**SEX**	1	1039	0	0.9654
		**txgroup**	1	1039	0	0.9817
		**log_Sum_anterior_chamber_baseline**	1	1039	1.45	0.2282
		**log_skin_density_baseline**	1	1039	1.83	0.1763
		**log_anterior_chamber_D4**	1	1039	0.23	0.6282
		**log_Sum_anterior_chamber_M1**	1	1039	0.02	0.8748
		**log_skin_density_M1**	1	1039	0	0.9814
		**log_Sum_anterior_chamber_M6**	1	1039	14.74	0.0001
		**log_skin_density_M6**	1	1039	0.89	0.3455
		**log_Sum_anterior_chamber_M12**	1	1039	601.24	<.0001
		**log_skin_density_M12**	1	1039	1.49	0.2232
		**log_skin_density_M18**	1	1039	0.06	0.7997

Table S5: Ocular medical history* of study participants by study area

Eye disorders (MedDRA coding)**	n (%)	n (%)	n (%)	
**Nord Kivu**	**Moxidectin (N=307)**	**Ivermectin (N=153)**	**Total (N=460)**	**p-value (b)**
Any eye disorder	225 (73.3%)	122 (79.7%)	347 (75.4%)	0.691
Amblyopia	4 (1.3%)	1 (0.7%)	5 (1.1%)	0.501
Asthenopia	2 (0.7%)	0 (0.0%)	2 (0.4%)	0.307
Blindness unilateral	1 (0.3%)	3 (2.0%)	4 (0.9%)	0.086
Cataract	1 (0.3%)	0 (0.0%)	1 (0.2%)	0.470
Conjunctivitis	4 (1.3%)	1 (0.7%)	5 (1.1%)	0.501
Corneal disorder	22 (7.2%)	15 (9.8%)	37 (8.0%)	0.419
Diplopia	1 (0.3%)	0 (0.0%)	1 (0.2%)	0.470
Eye discharge	1 (0.3%)	0 (0.0%)	1 (0.2%)	0.470
Eye irritation	1 (0.3%)	0 (0.0%)	1 (0.2%)	0.470
Eye pain	20 (6.5%)	10 (6.5%)	30 (6.5%)	0.914
Eye pruritus	126 (41.0%)	59 (38.6%)	185 (40.2%)	0.488
Eyelids pruritus	1 (0.3%)	1 (0.7%)	2 (0.4%)	0.639
Foreign body sensation in eyes	1 (0.3%)	3 (2.0%)	4 (0.9%)	0.086
Iridocyclitis	1 (0.3%)	0 (0.0%)	1 (0.2%)	0.470
Lacrimation increased	13 (4.2%)	11 (7.2%)	24 (5.2%)	0.231
Myopia	2 (0.7%)	0 (0.0%)	2 (0.4%)	0.307
Photophobia	2 (0.7%)	0 (0.0%)	2 (0.4%)	0.307
Pinguecula	2 (0.7%)	1 (0.7%)	3 (0.7%)	0.973
Presbyopia	36 (11.7%)	15 (9.8%)	51 (11.1%)	0.463
Pterygium	4 (1.3%)	1 (0.7%)	5 (1.1%)	0.501
Vision blurred	20 (6.5%)	8 (5.2%)	28 (6.1%)	0.524
Visual acuity reduced	92 (30.0%)	65 (42.5%)	157 (34.1%)	0.055
**Nord-Ituri**	**Moxidectin (N=315)**	**Ivermectin (N=157)**	**Total (N=472)**	**p-value (b)**
Any eye disorder	201 (63.8%)	107 (68.2%)	308 (65.3%)	0.815
Blindness	2 (0.6%)	3 (1.9%)	5 (1.1%)	0.223
Conjunctivitis	6 (1.9%)	3 (1.9%)	9 (1.9%)	0.958
Conjunctivitis allergic	1 (0.3%)	0 (0.0%)	1 (0.2%)	0.471
Diplopia	2 (0.6%)	3 (1.9%)	5 (1.1%)	0.223
Eye discharge	4 (1.3%)	1 (0.6%)	5 (1.1%)	0.504
Eye pain	16 (5.1%)	15 (9.6%)	31 (6.6%)	0.093
Eye pruritus	61 (19.4%)	27 (17.2%)	88 (18.6%)	0.485
Eye swelling	1 (0.3%)	0 (0.0%)	1 (0.2%)	0.471
Eyelid oedema	2 (0.6%)	0 (0.0%)	2 (0.4%)	0.308
Foreign body sensation in eyes	1 (0.3%)	3 (1.9%)	4 (0.8%)	0.085
Lacrimation increased	78 (24.8%)	42 (26.8%)	120 (25.4%)	0.844

Night blindness	0 (0.0%)	1 (0.6%)	1 (0.2%)	0.165
Ocular discomfort	1 (0.3%)	0 (0.0%)	1 (0.2%)	0.471
Ocular hyperaemia	3 (1.0%)	2 (1.3%)	5 (1.1%)	0.783
Photophobia	1 (0.3%)	3 (1.9%)	4 (0.8%)	0.085
Photopsia	1 (0.3%)	0 (0.0%)	1 (0.2%)	0.471
Presbyopia	8 (2.5%)	3 (1.9%)	11 (2.3%)	0.629
Scintillating scotoma	0 (0.0%)	1 (0.6%)	1 (0.2%)	0.165
Vision blurred	95 (30.2%)	48 (30.6%)	143 (30.3%)	0.878
Visual acuity reduced	38 (12.1%)	14 (8.9%)	52 (11.0%)	0.267
Vitreous floaters	2 (0.6%)	0 (0.0%)	2 (0.4%)	0.308
**Lofa**	**Moxidectin (N=200)**	**Ivermectin (N= 99)**	**Total (N=299)**	**p-value (b)**
Any eye disorder	180 (90.0%)	89 (89.9%)	269 (90.0%)	0.725
Arcus lipoides	1 (0.5%)	0 (0.0%)	1 (0.3%)	0.492
Blepharospasm	1 (0.5%)	0 (0.0%)	1 (0.3%)	0.492
Blindness unilateral	5 (2.5%)	1 (1.0%)	6 (2.0%)	0.418
Cataract	26 (13.0%)	18 (18.2%)	44 (14.7%)	0.205
Chorioretinal atrophy	1 (0.5%)	0 (0.0%)	1 (0.3%)	0.492
Chorioretinal disorder	2 (1.0%)	1 (1.0%)	3 (1.0%)	0.963
Chorioretinitis	12 (6.0%)	9 (9.1%)	21 (7.0%)	0.288
Conjunctival discolouration	1 (0.5%)	1 (1.0%)	2 (0.7%)	0.587
Conjunctival hyperaemia	5 (2.5%)	2 (2.0%)	7 (2.3%)	0.842
Conjunctivitis	3 (1.5%)	1 (1.0%)	4 (1.3%)	0.762
Conjunctivitis allergic	2 (1.0%)	0 (0.0%)	2 (0.7%)	0.331
Corneal lesion	1 (0.5%)	0 (0.0%)	1 (0.3%)	0.492
Diplopia	1 (0.5%)	0 (0.0%)	1 (0.3%)	0.492
Eye discharge	3 (1.5%)	1 (1.0%)	4 (1.3%)	0.762
Eye disorder	1 (0.5%)	0 (0.0%)	1 (0.3%)	0.492
Eye irritation	8 (4.0%)	3 (3.0%)	11 (3.7%)	0.732
Eye movement disorder	1 (0.5%)	0 (0.0%)	1 (0.3%)	0.492
Eye pain	15 (7.5%)	4 (4.0%)	19 (6.4%)	0.301
Eye pruritus	88 (44.0%)	53 (53.5%)	141 (47.2%)	0.149
Eyelid ptosis	3 (1.5%)	0 (0.0%)	3 (1.0%)	0.234
Eyelids pruritus	2 (1.0%)	1 (1.0%)	3 (1.0%)	0.963
Foreign body sensation in eyes	3 (1.5%)	4 (4.0%)	7 (2.3%)	0.155
Glaucoma	77 (38.5%)	31 (31.3%)	108 (36.1%)	0.443
Halo vision	3 (1.5%)	1 (1.0%)	4 (1.3%)	0.762
Iris adhesions	2 (1.0%)	0 (0.0%)	2 (0.7%)	0.331
Iris atrophy	1 (0.5%)	2 (2.0%)	3 (1.0%)	0.199
Iritis	6 (3.0%)	2 (2.0%)	8 (2.7%)	0.668
Lacrimation increased	19 (9.5%)	10 (10.1%)	29 (9.7%)	0.780

Lens disorder	0 (0.0%)	1 (1.0%)	1 (0.3%)	0.146
Macular degeneration	6 (3.0%)	1 (1.0%)	7 (2.3%)	0.313
Macular oedema	0 (0.0%)	1 (1.0%)	1 (0.3%)	0.146
Myopia	1 (0.5%)	0 (0.0%)	1 (0.3%)	0.492
Ocular hyperaemia	1 (0.5%)	2 (2.0%)	3 (1.0%)	0.199
Ocular hypertension	1 (0.5%)	0 (0.0%)	1 (0.3%)	0.492
Optic atrophy	0 (0.0%)	1 (1.0%)	1 (0.3%)	0.146
Papilloedema	1 (0.5%)	0 (0.0%)	1 (0.3%)	0.492
Periorbital oedema	1 (0.5%)	0 (0.0%)	1 (0.3%)	0.492
Photophobia	1 (0.5%)	0 (0.0%)	1 (0.3%)	0.492
Presbyopia	11 (5.5%)	1 (1.0%)	12 (4.0%)	0.077
Pterygium	5 (2.5%)	1 (1.0%)	6 (2.0%)	0.418
Retinal degeneration	5 (2.5%)	2 (2.0%)	7 (2.3%)	0.842
Retinal exudates	0 (0.0%)	3 (3.0%)	3 (1.0%)	0.012*
Retinal haemorrhage	1 (0.5%)	0 (0.0%)	1 (0.3%)	0.492
Retinitis	1 (0.5%)	1 (1.0%)	2 (0.7%)	0.587
Ulcerative keratitis	2 (1.0%)	0 (0.0%)	2 (0.7%)	0.331
Uveitis	1 (0.5%)	1 (1.0%)	2 (0.7%)	0.587
Vision blurred	2 (1.0%)	0 (0.0%)	2 (0.7%)	0.331
Visual impairment	134 (67.0%)	59 (59.6%)	193 (64.5%)	0.641
Vitreous floaters	1 (0.5%)	0 (0.0%)	1 (0.3%)	0.492
**Nkwanta**	**Moxidectin (N=158)**	**Ivermectin (N= 83)**	**Total (N=241)**	**p-value (b)**
Any eye disorder	61 (38.6%)	28 (33.7%)	89 (36.9%)	0.481
Diplopia	1 (0.6%)	0 (0.0%)	1 (0.4%)	0.463
Exophthalmos	1 (0.6%)	0 (0.0%)	1 (0.4%)	0.463
Eye pain	4 (2.5%)	2 (2.4%)	6 (2.5%)	0.931
Eye pruritus	12 (7.6%)	4 (4.8%)	16 (6.6%)	0.396
Lacrimation increased	3 (1.9%)	0 (0.0%)	3 (1.2%)	0.203
Ocular hyperaemia	1 (0.6%)	0 (0.0%)	1 (0.4%)	0.463
Vision blurred	46 (29.1%)	23 (27.7%)	69 (28.6%)	0.758

* considered relevant by investigators and entered into the data base, **across all participants, (b) p-value for Chi-Square.

Table S6: Output of final logistic model of the factors impacting the risk to have at least one ocular Mazzotti reaction

Type III Tests of Fixed Effects				
**Effect**	**Num DF**	**Den DF**	**F Value**	**p-value**
**Sex**	1	1451	6.21	0.0128
**Treatment group**	1	1451	2.52	0.1127
**MfAC Infection level at Screening**	3	1451	2.46	0.0608
**MfAC _Infection level at D4**	3	1451	1.47	0.2219

Odds Ratio Estimates									
**Effect**	**Effect categories**	**Beta Coef.**	**Standard Error**	**DF**	**t Value**	**p-value**	**OR**	**95% Confidence Limits of OR**	
**Sex**	**Male**	**0**					**1**		
	**Female**	0.43	0.1726	1451	2.49	0.0128	1.537	1.096	2.157
**Treatment**	**Moxidectin**	**0**					1		
	**Ivermectin**	-0.2929	0.1845	1451	-1.59	0.1127	0.746	0.519	1.072
**MfAC Infection level at Screening**	**0 mfACs**	**0**					1		
	**> 0 mfACs - <= 5 mfACs**	0.04948	0.2407	1451	0.21	0.8372	1.051	0.655	1.685
	**> 5 mfACs - <= 10 mfACs**	0.3968	0.392	1451	1.01	0.3116	1.487	0.689	3.208
	**> 10 mfACs**	0.9947	0.3846	1451	2.59	0.0098	2.704	1.272	5.749
**MfAC Infection level at D4**	**0 mfACs**	0					1		
	**> 0 mfACs - <= 5 mfACs**	0.4177	0.2168	1451	1.93	0.0542	1.518	0.992	2.323
	**> 5 mfACs - <= 10 mfACs**	0.466	0.376	1451	1.24	0.2154	1.594	0.762	3.332
	**> 10 mfACs**	0.482	0.3598	1451	1.34	0.1805	1.619	0.8	3.28

Ocular Mazzotti reactions started within 1 month of treatment

Table S7: Number (%) of participants with ocular adverse events starting within 1 and between month 1 and end of 6 months after treatment by ocular AE based on MedDRA coding

		Starting within 1 month of treatment				Starting between month 1 and end of month 6			
		Moxidectin		Ivermectin		Moxidectin		Ivermectin	
*Number (%) of participants with at least one ocular adverse event		223	22.9	82	16.7	92	9.5	43	8.8
**MedDRA High Level Group Term**	**MedDRA Preferred Term**	n	**%***	n	**%***	n	**%***	n	**%***
Anterior eye structural change, deposit and degeneration	Cataract							1	0.2
	Corneal erosion					1	0.1		
	Iris atrophy	1	0.1						
	Pterygium	1	0.1			1	0.1	2	0.4
Eye disorders nec	Eye pain	47	4.8	12	2.4	30	3.1	10	2.0
	Eye swelling	1	0.1						
	Eyelid disorder	2	0.2	2	0.4				
	Eyelid pain	1	0.1						
	Eyelids pruritus	1	0.1			1	0.1		
	Keratoconjunctivitis sicca					1	0.1		
	Lacrimation increased	6	0.6	8	1.6	4	0.4		
	Ocular discomfort	5	0.5	2	0.4				
Glaucoma and ocular hypertension	Glaucoma	2	0.2	1	0.2	1	0.1		
	Ocular hypertension	1	0.1						
Ocular infections, irritations and inflammations	Blepharitis			1	0.2				
	Chalazion							1	0.2
	Conjunctival hyperaemia	8	0.8	1	0.2				
	Conjunctivitis	33	3.4	13	2.7	14	1.4	4	0.8
	Conjunctivitis allergic	5	0.5	4	0.8	9	0.9	5	1.0
	Cyclitis					1	0.1		
	Episcleritis							1	0.2
	Eye discharge	2	0.2	2	0.4	1	0.1		
	Eye inflammation			1	0.2				
	Eye irritation	1	0.1	2	0.4			1	0.2

		Starting within 1 month of treatment				Starting between month 1 and end of month 6			
		Moxidectin		Ivermectin		Moxidectin		Ivermectin	
	Eye pruritus	49	5.0	13	2.7	12	1.2	12	2.4
	Eyelid oedema	19	2.0	5	1.0	2	0.2		
	Iritis	3	0.3						
	Keratitis			1	0.2	2	0.2		
	Ocular hyperaemia	8	0.8	1	0.2	2	0.2		
	Uveitis			1	0.2			1	0.2
Ocular sensory symptoms nec	Abnormal sensation in eye	2	0.2	1	0.2	1	0.1	1	0.2
	Foreign body sensation in eyes	5	0.5	3	0.6	1	0.1	3	0.6
	Photophobia	2	0.2	1	0.2				
Ocular structural change, deposit and degeneration nec	Macular degeneration	1	0.1						
	Macular hole					1	0.1		
	Maculopathy	1	0.1						
	Vitreous floaters	1	0.1						
Vision disorders	Photopsia	1	0.1	1	0.2				
	Vision blurred	8	0.8	4	0.8	1	0.1		
	Visual acuity reduced	1	0.1			1	0.1		
	Visual impairment	5	0.5	2	0.4	5	0.5	1	0.2

* percentage calculated based on total number of individuals treated who had both eyes evaluated (Moxidectin N=973, Ivermectin N=490)

Table S8: Number of ocular adverse events starting within 1 and between month 1 and end of 6 months after treatment by ocular AE based on MedDRA coding

		Starting within 1 month of treatment				Starting between month 1 and end of month 6			
		Moxidectin		Ivermectin		Moxidectin		Ivermectin	
Total Number of ocular adverse events		230		90		96		45	
**MedDRA High Level Group Term**	**MedDRA Preferred Term**	n	%*	n	%*	n	%*	n	%*
Anterior Eye Structural Change, Deposit And Degeneration	Cataract							1	0.7
	Corneal Erosion					1	0.7		
	Iris Atrophy	1	0.3						
	Pterygium	1	0.3			1	0.7	2	1.4
Eye Disorders Nec	Eye Pain	49	15.3	12	3.8	33	23.4	11	7.8
	Eye Swelling	1	0.3						
	Eyelid Disorder	2	0.6	2	0.6				
	Eyelid Pain	1	0.3						
	Eyelids Pruritus	1	0.3			1	0.7		
	Keratoconjunctivitis Sicca					1	0.7		
	Lacrimation Increased	8	2.5	10	3.1	4	2.8		
	Ocular Discomfort	5	1.6	2	0.6				
Glaucoma And Ocular Hypertension	Glaucoma	2	0.6	1	0.3	1	0.7		
	Ocular Hypertension	1	0.3						
Ocular Infections, Irritations And Inflammations	Blepharitis			1	0.3				
	Chalazion							1	0.7
	Conjunctival Hyperaemia	8	2.5	1	0.3				
	Conjunctivitis	33	10.3	13	4.1	14	9.9	4	2.8
	Conjunctivitis Allergic	5	1.6	5	1.6	9	6.4	5	3.5
	Cyclitis					1	0.7		
	Episcleritis							1	0.7
	Eye Discharge	2	0.6	2	0.6	1	0.7		
	Eye Inflammation			1	0.3				
	Eye Irritation	1	0.3	2	0.6			1	0.7

		Starting within 1 month of treatment				Starting between month 1 and end of month 6			
		Moxidectin		Ivermectin		Moxidectin		Ivermectin	
	Eye Pruritus	52	16.3	16	5.0	13	9.2	13	9.2
	Eyelid Oedema	19	5.9	5	1.6	2	1.4		
	Iritis	3	0.9						
	Keratitis			1	0.3	2	1.4		
	Ocular Hyperaemia	8	2.5	1	0.3	2	1.4		
	Uveitis			1	0.3			1	0.7
Ocular Sensory Symptoms Nec	Abnormal Sensation In Eye	2	0.6	1	0.3	1	0.7	1	0.7
	Foreign Body Sensation In Eyes	5	1.6	5	1.6	1	0.7	3	2.1
	Photophobia	2	0.6	1	0.3				
Ocular Structural Change, Deposit And Degeneration Nec	Macular Degeneration	1	0.3						
	Macular Hole					1	0.7		
	Maculopathy	1	0.3						
	Vitreous Floaters	1	0.3						
Vision Disorders	Photopsia	1	0.3	1	0.3				
	Vision Blurred	8	2.5	4	1.3	1	0.7		
	Visual Acuity Reduced	1	0.3			1	0.7		
	Visual Impairment	5	1.6	2	0.6	5	3.5	1	0.7

* percentage calculated based on total number of ocular adverse events across treatment groups within the relevant time period. MedDRA Medical dictionary for regulatory activities

Table S9: Output of final logistic model of the factors and covariates impacting the risk to have at least one ocular adverse event

Type III Tests of Fixed Effects				
Effect	Num DF	Den DF	F Value	p-value
Age	1	1445	4.63	0.0315
Sex	1	1445	5.94	0.015
Treatment group	1	1445	5.76	0.0165
MfAC Infection level at M1	3	1445	5.91	0.0005

Odds Ratio Estimates									
Effect	Effect categories	Beta	Standard Error	DF	t Value	p-value	OR	95% Confidence Limits of OR	
Age	41.924*	0					1		
	42.924*	0.00962	0.00447	1445	2.15	0.0315	1.01	1.001	1.019
Sex	Male	0					1		
	Female	0.3394	0.1393	1445	2.44	0.015	1.404	1.068	1.845
Treatment	Moxidectin	0					1		
	Ivermectin	-0.3436	0.1431	1445	-2.4	0.0165	0.709	0.536	0.939
MfAC Infection level at M1	0 mfACs	0					1		
	> 0 mfACs - <= 5 mfACs	0.1914	0.1747	1445	1.1	0.2734	1.211	0.86	1.706
	> 5 mfACs - <= 10 mfACs	0.5003	0.3489	1445	1.43	0.1518	1.649	0.832	3.27
	> 10 mfACs	1.0709	0.2643	1445	4.05	<.0001	2.918	1.737	4.9

*mean age as reference “category” and mean age + 1 to evaluate the OR associated with an increase in age of 1 unit i.e. one year.

Table S10: Literature data on mfAC number change early after treatment with diethylcarbamazine, ivermectin and suramin

Study Location<br>Treatments<br>(number treated)	Pre-Treatment<br>SmfD[1]	Pre-treatment ocular<br>microfilariae	[post-treatment mfAC measurement]<br>mfAC data/changes early after treatment	Ref
Liberia<br>DEC oral (10)<br>DEC topical (10)	GM mf/snip<br>14.1<br>16.7	 0<br>0	[1, 3, 7, and 14 days, 2 and 6 months]<br>Increase in number of individuals with intra-ocular mf to Day 14<br>Increase in mfAC mean to day 7 oral DEC, to day 14 topical DEC	(1)
Senegal<br>IVM 5 µg/kg (8)<br>IVM 10 µg/kg (8)<br>IVM 30 µg/kg (8)<br>IVM 50 µg/kg (8)		Not reported	[Daily examination with slit lamp to day 28]<br>No ocular data reported	(2)
Ghana<br>IVM 50 µg/kg (3)<br>IVM 100 µg/kg (5)<br>IVM 150 µg/kg (5)<br>IVM 200 µg/kg (6)	GM mf/mg<br>80.3<br>79.5<br>76<br>64.5	GM (95% CI) for participants with mfAC PreTx:<br>≈5 (≈2.5 - ≈ 9) (read from Fig 1)	[Day 2, 4, 8, 14, 28, months 3 and 6]<br>• For participants with ocular mf pre-Tx: mobilisation of mf into the AC, reaching peak at Day 4, slow mfAC elimination over 3 months<br>• 6 participants with no ocular mf pre-treatment had ocular mf at various times between days 2 and 14.	(3, 4)
France<br>IVM 150 µg/kg (5)<br>IVM 200 µg/kg (7)		9 with ocular involvement, no details reported	[thorough ophthalmological examination before treatment and periodically during 2 weeks in hospital after treatment]<br>Among individuals treated with 200 µg/kg<br>• mfAC in one participant on Day 3 but not on day 13<br>• mfAC present pre-treatment not seen on Day 2 but on Day 19<br>• mfAC present pre-treatment not observed on Day 33.	(5)
Liberia<br>IVM 12 mg (10)<br>DEC (10)<br>Placebo (10)	GM mf/mg<br>47.3<br>35.6<br>43.6	mfAC present – mfCOR present<br>9/10 – 7/10<br>9/10 – 8/10<br>7/10 – 8/10	[Days 2, 4, 8, and 14, months 3, 6, 12]<br>• IVM: mfAC GM increase on Days 2 and 4 (Fig 8 (6)), not statistically significant, resulting in statement that mfAC did not change significantly during first 2 weeks, but was significantly reduced at month 3 and 6.<br>• DEC, mfAC number fell after Day 2 to level significantly lower than in placebo group on Day 14<br>• Plac: mfAC no change	(6, 7)

Study Location Treatments (number treated)	Pre-Treatment SmfD[1]	Pre-treatment ocular microfilariae	[post-treatment mfAC measurement]<br>mfAC data/changes early after treatment	Ref
Mali<br>IVM 12 mg (10)<br>DEC (10)<br>Placebo (10)	Mean±SD<br>130.4±71.8<br>100.3±48.2<br>99.4±52.4	mfAC present<br>8/10<br>5/10<br>9/10	[Days 2, 4, 8, 14, 28, months 3, 6, 12]<br>• IVM: mfAC present: Day 4 9/10, Day 14 7/10, Day 28 7/10<br>• DEC: mfAC present: Day 4 2/10, Day 14 4/10, Day 28 2/10<br>• Plac: mfAC present: Day 4 8/10, Day 14 9/10, Day 28 5/10	(8)
Ghana (OCP area)<br>Placebo (17)<br>DEC (19)<br>IVM 12 mg (18)	GM (range) mf/mg<br>137 (61-427)<br>113 (38-1419)<br>153 (38-1419)	mfAC present - mfCor present<br>16/17 – 3/17<br>17/19 – 3/19<br>17/18 – 6/18<br>Subgroup ocular data focussed analysis<br>Tx (n), GM mfAC - GM mfCOR (Fig 2, 3, (9))<br>Placebo (16), ≈2.5 - ≈0.2<br>DEC (17), ≈ 4 - ≈0.08<br>IMV (15), ≈ 6 - ≈0.48	[Day 2, 4, 8, 14, 28, months 3, 6, 12]<br>• DEC: near complete mfAC elimination by Day 8 with increase in GM mfCOR to ≈ 0.68 on Day 4 prior to elimination<br>• IVM: significant increase in GM mfAC to 8 on Day 2 and 11 on Day with subsequent slow reduction to near absence by month 6, increase in GM mfCOR to ≈ 0.9 on Day 4<br>Ocular data focussed analysis (9) excluded participants without month 3 and 6 evaluations	(10) (9)
Senegal<br>Placebo (17)<br>DEC (19)<br>IVM 12 mg (180-260 µg/kg) (18)	GM mf/mg<br>51.0<br>44.5<br>34.0	mfAC present - mfCor present<br>6 - 3<br>4 - 0<br>1 - 1	[Day 2, 4, 8, 14, 28, months 3 and 6]<br>Placebo<br>• mfAC pre-Tx in 6 participants still present at Month 6.<br>DEC:<br>• mfAC PreTx in 4 participants not found after Day 4<br>• mfAC that had appeared after Tx in one patient no longer present after 3 months<br>Ivermectin:<br>• mfAC that had appeared after ivermectin in 4 patients no longer present in 3 patients at 6 months<br>• mfAC and mfCOR present before ivermectin not present at 3 and 6 months	(11)
Liberia<br>Placebo (48)<br>IVM 100 µg/kg (49)<br>IVM 150 µg/kg (52)<br>IVM 200 µg/kg (51)	GM mf/mg (95% CI)<br>21.0 (17.1-25.7)<br>17.9 (14.5-22.1)<br>21.2 (16.7-26.8)<br>20.8 (16.4-26.1)	Not provided (included in 'ocular disease index' which includes number of mfCOR, number of PCO and severity of uveitis and limbitis)	[Day 3, months 3, 6, and 12]<br>No details provided - included in 'ocular disease index' which includes number of mfCOR, number of PCO and severity of uveitis and limbitis	(12)

Study Location Treatments (number treated)	Pre-Treatment SmfD[1]	Pre-treatment ocular microfilariae	[post-treatment mfAC measurement] mfAC data/changes early after treatment	Ref
Liberia<br>DEC oral (11)<br>DEC lotion I (11)<br>DEC lotion II (8)<br>IVM 150 µg/kg (10)<br>Placebo (10)	Mean mf/mg (SE)<br>63 (36-90)<br>58 (38-78)<br>48 (15-81)<br>44 (17-71)<br>42 (23-61)	mfAC – mfCOR GM<br>Not provided - <1<br>Not provided - <1<br>Not provided - <1<br>Not provided - <1<br>Not provided - <1	[Days 3, 14, months 2 and 10]<br>mfAC: No quantitative information provided: in all treatment groups mfAC fell rapidly with treatment, significant difference to pre-Tx at month 2	(13)
Ghana (OCP area)<br>Placebo (38)<br>IVM 100 µg/kg (39)<br>IVM 150 µg/kg (42)<br>IVM 200 µg/kg (35)	GM mf/mg (range)<br>121.9 (94.3-157.6)<br>141.1 (103.1-195.3)<br>102.2 (77.4-134.8)<br>114.9 (81.2-162.4)	mfAC present (GM)– mfCOR present<br>30/39 – 8/39<br>30/38 – 8/38<br>35/39 – 7/42<br>28/35 – 5/35	[Day 3 or 4, months 3, 6, 12]<br>• Plac: mfAC gradual decrease from GM ≈3.9 PreTx to ≈1.5 Month 6<br>• IVM 100: mfAC GM ≈6.2 preTx, ≈6.15 Day 4, ≈0.5 Month 6<br>• IVM 150: mfAC GM ≈5.8 preTx, ≈6.5 Day 4, ≈0.5 Month 6<br>• IVM 200: mfAC GM ≈6.1 preTx, ≈5.9 Day 4, ≈0.5 Month 6	(9)<br>Subgroup of participants in (14)
Côte d'Ivoire<br>Placebo (53)<br>IVM 100 µg/kg (55)<br>IVM 150 µg/kg (55)<br>IVM 200 µg/kg (51)	GM mf/mg<br>64.3<br>58.5<br>62.8<br>60.9	n (%) with mfAC – with mfCOR<br>37/53 (69.8) – 24/53 (45.3)<br>38/54 (70.4) – 15/54 (31.5)<br>33/53 (70.4) – 14/53 (26.4)<br>37/55 (67.3) – 18/55 (32.7)	[Day 4, month 3, 6]]<br>n (%) mfAC Day 4: Plac 40/53<br>IVM 100 : 44/54 (81.5)<br>IVM 150 : 39/53 (73.6)<br>IVM 200 : 40/55 (72.7)	(15)
Liberia<br>Placebo (48)<br>IVM 100 µg/kg (49)<br>IVM 150 µg/kg (52)<br>IVM 200 µg/kg (51)	GM mf/mg<br>21.0<br>17.9<br>21.2<br>20.8	n (%) with mfAC – with mfCOR<br>26 (54.2) – 16 (33.3)<br>19 (38.8) – 8 (16.3)<br>27 (51.9) – 15 (28.8)<br>24 (27.1) – 14 (27.5)	[Day 3, Months 3, 6, 12]<br>GM mfAC decrease in all treatment groups to Day 3<br>Abnormal motility of mfAC during the first 3 days post IVM ("extended configuration, periodic immobility, occasional unusual motion in which the mf bent only in the middle suggestive of a spastic paralysis")	(16)
Liberia<br>Placebo (9)<br>IVM 100 µg/kg (10)<br>IVM 150 µg/kg (8)<br>IVM 200 µg/kg (12)	GM mf/mg<br>32.14<br>33.47<br>33.47<br>38.65	GM mfAC – GM mfCOR<br>20-54 – 1.1<br>17.91 – 0.7<br>34.87 – 1.14<br>17.17 – 0.99	[Day 3, months 3, 6, 12, 18, 24, and 36]<br>No increase in GM or % of individuals with mfAC reported	(17)
Côte d'Ivoire<br>IVM 150 µg/kg (103)<br>(6-<14 yrs)	Mean (range)<br>36.1 mf/mg (10.2 – 265.1)	n (%) with mfAC – with mfCOR<br>34 (33) – 40 (38.8)	[Day 4, month 3, 6]<br>n (%) with mfAC: Day 4 55 (53.4)<br>increase in n from treatment significant (p<0.01) and in number of mfAC in those with mfAC (p<0.001<br>n (%) with mfAC: Month 3 9 (9.1)	(18)

Study Location Treatments (number treated)	Pre-Treatment SmfD[1]	Pre-treatment ocular microfilariae	[post-treatment mfAC measurement] mfAC data/changes early after treatment	Ref
Ghana<br>Suramin (20)	GM (95%CI) mf/mg<br>231 (166-320)	Presence - GM (95% CI)<br>mfAC 10/20 - 8.1 (4.0-15.6)<br>mfCOR 7/20<br>CPO 10/20	[not specified, data presented include 1, 2, 3, 4, 5, 6, 13, 26, 52 104 weeks after treatment]<br>• No significant change until Week 5.<br>• mfAC GM (95% CI) 1 - 2 - 3 – 4 weeks after suramin: 6.3 (1.9-17.2) – 7.7 (2.2-23.0) – 7.0 (2.1-19.9) – 5.4 (1.5-15.2)	(19)
Ghana<br>IVM 150 µg/kg +Plac 7 days later (31)<br>IVM 150 µg/kg +Alb 800 mg 7 days later (34)	GM (95%CI) mf/mg<br>394 (340-458)<br><br>338 (294-388)	Presence - GM (95% CI)<br>mfAC 28/31 – 12.3 (6.7–21.9)<br>DmfCOR 5/31 – 0.2 (0.02-0.48)<br>mfAC 28/34 – 7.2 (4.2-12.5)<br>DmfCOR 4/34 – 0.14 (0.04–0.35)	[Day 4, 7, 11, 18, 30, 90, 180, 270, 364]<br>Day 4 data not reported and no mention of mfAC peak in the text<br>mfAC GM (95% CI), 1 - 1.6 - 2.6 - 4 weeks post-Tx<br>IVM+Plac:<br>12.2 (6.7-21.9) – 11.3 (6.1-20.4) – 8.2 (4.3-15.2) – 4.0 (1.8-7.9)<br>IVM+ Alb:<br>6.6 (4.0-10.5) – 5.8 (4.0-4.0) – 2.8 (1.6-4.6) – 1.1 (0.6-1.9)<br>Persistance of mfAC (1-12) to one year in 12 and 8 participants treated with IVM+Plac and IVM+Alb, respectively	(20)
Ghana<br>IVM 12 mg+Plac (14)<br>Alb 400 mg +Plac(14)<br>IVM 12 mg +Alb 400 mg (14)	GM (95%CI) mf/mg<br>148.5 (118.6-185.9)<br>151.2 (117-194.0)<br>196.6 (135.5-285.0)	Presence [mean (range)]<br>mfAC<br>7/14 [0.8 (1-4)] –<br>11/14 [4.1 (1-14)–<br>8/14 [5.5 (1-24)<br>mfCOR/CPO<br>0/14<br>2/14<br>2/14	[Day 4, 8, 30, 180, 270, 365]<br>No data reported for time points before Day 365.	(21)

Alb albendazole, CPO corneal punctate opacities, DEC diethylcarbamazine, DmfCOR dead microfilariae in the cornea, GM geometric mean, IVM ivermectin, mf microfilariae, mfAC microfilariae in the anterior chamber, mfCOR microfilariae in the cornea, Plac placebo , Tx treatment

[1] values across studies are not necessarily comparable due to differences in methods of skin microfilariae detection.

Table S11: Microfilariae in the blood and urine before and after treatment (data from the moxidectin Phase 2 study, across all participants and for participants with > 20mf/mg skin)

	2 mg moxidectin					4 mg moxidectin					8 mg moxidectin					150 µg/kg ivermectin				
	N	Mean	SD	Min	Max	N	Mean	SD	Min	Max	N	Mean	SD	Min	Max	N	Mean	SD	Min	Max
All participants																				
mf/ml blood																				
PreTx	44	0	0	0	0	45	0.04	0.30	0	2.00	38	0.03	0.16	0	1.00	45	0.04	0.30	0	2.00
Day 1	44	0.05	0.30	0	2.00	45	0	0	0	0	38	0	0	0	0	45	0	0	0	0
Day 2	44	0.14	0.67	0	4.00	45	0.53	1.31	0	6.00	38	0	0	0	0	45	0.18	0.72	0	4.00
Day 4	44	0.14	0.51	0	2.00	45	0.22	0.77	0	4.00	38	0	0	0	0	45	0.44	1.65	0	10
Day 8	44	0.27	1.02	0	6.00	45	0.22	1.22	0	8.00	38	0	0	0	0	45	0.58	2.28	0	14.00
Day 13	44	0.14	0.51	0	2.00	45	0	0	0	0	38	0	0	0	0	45	0.13	0.66	0	4.00
Day 18	44	0	0	0	0	45	0	0	0	0	38	0	0	0	0	45	0.09	0.60	0	4.00
Month 1	44	0	0	0	0	45	0	0	0	0	38	0	0	0	0	45	0	0	0	0
Month 2	43	0	0	0	0	45	0	0	0	0	38	0	0	0	0	45	0	0	0	0
Month 3	44	0	0	0	0	45	0	0	0	0	38	0	0	0	0	44	0	0	0	0
Month 6	44	0	0	0	0	45	0	0	0	0	38	0	0	0	0	44	0	0	0	0
Month 12	43	0	0	0	0	45	0	0	0	0	37	0.03	0.16	0	1.00	43	0	0	0	0
Month 18	42	0	0	0	0	45	0	0	0	0	37	0	0	0	0	42	0	0	0	0
mf/10 ml urine																				
PreTx	44	0	0	0	0	45	0.07	0.33	0	2.00	38	0	0	0	0	45	0	0	0	0
Day 1	44	0	0	0	0	45	0	0	0	0	38	0	0	0	0	45	0	0	0	0
Day 2	44	0	0	0	0	45	0	0	0	0	38	0	0	0	0	45	0	0	0	0
Day 4	44	0.30	1.81	0	12.00	45	0	0	0	0	38	0	0	0	0	45	0	0	0	0
Day 8	44	0.11	0.49	0	3.00	45	0	0	0	0	38	0	0	0	0	45	0	0	0	0
Day 13	44	0.07	0.33	0	2.00	45	0	0	0	0	38	0	0	0	0	45	0	0	0	0
Day 18	44	0.09	0.42	0	2.00	45	0	0	0	0	38	0	0	0	0	45	0	0	0	0
Month 1	44	0	0	0	0	45	0	0	0	0	38	0	0	0	0	45	0.02	0.15	0	1.00
Month 2	43	0	0	0	0	45	0	0	0	0	38	0	0	0	0	45	0	0	0	0
Month 3	44	0	0	0	0	45	0	0	0	0	38	0	0	0	0	44	0	0	0	0
Month 6	44	0	0	0	0	45	0	0	0	0	38	0	0	0	0	44	0	0	0	0
Month 12	43	0	0	0	0	45	0	0	0	0	37	0.05	0.23	0	1.00	43	0	0	0	0
Month 18	42	0	0	0	0	45	0	0	0	0	37	0	0	0	0	42	0	0	0	0

	2 mg moxidectin					4 mg moxidectin					8 mg moxidectin					150 µg/kg ivermectin				
	N	Mean	SD	Min	Max	N	Mean	SD	Min	Max	N	Mean	SD	Min	Max	N	Mean	SD	Min	Max
Participants with >20 mf/mg skin																				
mf/ml blood																				
PreTx	23	0	0	0	0	23	0.09	0.42	0	2.00	15	0.07	0.26	0	1.00	21	0.10	0.44	0	2.00
Day 1	23	0.09	0.42	0	2.00	23	0	0	0	0	15	0	0	0	0	21	0	0	0	0
Day 2	23	0.26	0.92	0	4.00	23	0.70	1.55	0	6.00	15	0	0	0	0	21	0.29	0.96	0	4.00
Day 4	23	0.26	0.69	0	2.00	23	0.35	0.98	0	4.00	15	0	0	0	0	21	0.95	2.33	0	10
Day 8	23	0.52	1.38	0	6.00	23	0.43	1.70	0	8.00	15	0	0	0	0	21	1.24	3.25	0	14.00
Day 13	23	0.26	0.69	0	2.00	23	0	0	0	0	15	0	0	0	0	21	0.29	0.96	0	4.00
Day 18	23	0	0	0	0	23	0	0	0	0	15	0	0	0	0	21	0.19	0.87	0	4.00
Month 1	23	0	0	0	0	23	0	0	0	0	15	0	0	0	0	21	0	0	0	0
Month 2	22	0	0	0	0	23	0	0	0	0	15	0	0	0	0	21	0	0	0	0
Month 3	23	0	0	0	0	23	0	0	0	0	15	0	0	0	0	20	0	0	0	0
Month 6	23	0	0	0	0	23	0	0	0	0	15	0	0	0	0	20	0	0	0	0
Month 12	22	0	0	0	0	23	0	0	0	0	15	0	0	0	0	20	0	0	0	0
Month 18	22	0	0	0	0	23	0	0	0	0	15	0	0	0	0	20	0	0	0	0
mf/10 ml urine																				
PreTx	23	0	0	0	0	23	0.13	0.46	0	2.00	15	0	0	0	0	21	0.10	0.44	0	2.00
Day 1	23	0	0	0	0	23	0	0	0	0	15	0	0	0	0	21	0	0	0	0
Day 2	23	0	0	0	0	23	0	0	0	0	15	0	0	0	0	21	0	0	0	0
Day 4	23	0.57	2.50	0	12.00	23	0	0	0	0	15	0	0	0	0	21	0	0	0	0
Day 8	23	0.22	0.67	0	3.00	23	0	0	0	0	15	0	0	0	0	21	0	0	0	0
Day 13	23	0.13	0.46	0	2.00	23	0	0	0	0	15	0	0	0	0	21	0	0	0	0
Day 18	23	0.17	0.58	0	2.00	23	0	0	0	0	15	0	0	0	0	21	0	0	0	0
Month 1	23	0	0	0	0	23	0	0	0	0	15	0	0	0	0	21	0.05	0.22	0	1.00
Month 2	22	0	0	0	0	23	0	0	0	0	15	0	0	0	0	21	0	0	0	0
Month 3	23	0	0	0	0	23	0	0	0	0	15	0	0	0	0	20	0	0	0	0
Month 6	23	0	0	0	0	23	0	0	0	0	15	0	0	0	0	20	0	0	0	0
Month 12	22	0	0	0	0	23	0	0	0	0	15	0	0	0	0	20	0	0	0	0
Month 18	22	0	0	0	0	23	0	0	0	0	15	0	0	0	0	20	0	0	0	0

REFERENCES

1. Taylor HR, Greene BM. Ocular changes with oral and transepidermal diethylcarbamazine therapy of onchocerciasis. Br J Ophthalmol. 1981;65(7):494-502.

2. Aziz MA, Diallo S, Diop IM, Lariviere M, Porta M. Efficacy and tolerance of ivermectin in human onchocerciasis. Lancet. 1982;2(8291):171-3.

3. Awadzi K, Dadzie KY, Schulz-Key H, Haddock DR, Gilles HM, Aziz MA. Ivermectin in onchocerciasis. Lancet. 1984;2(8408):921.

4. Awadzi K, Dadzie KY, Shulz-Key H, Haddock DR, Gilles HM, Aziz MA. The chemotherapy of onchocerciasis X. An assessment of four single dose treatment regimes of MK-933 (ivermectin) in human onchocerciasis. Ann Trop Med Parasitol. 1985;79(1):63-78.

5. Coulaud JP, Lariviere M, Aziz MA, Gervais MC, Gaxotte P, Deluol AM, et al. Ivermectin in onchocerciasis. Lancet. 1984;2(8401):526-7.

6. Taylor HR, Murphy RP, Newland HS, White AT, D'Anna SA, Keyvan-Larijani E, et al. Treatment of onchocerciasis. The ocular effects of ivermectin and diethylcarbamazine. Arch Ophthalmol. 1986;104(6):863-70.

7. Greene BM, Taylor HR, Cupp EW, Murphy RP, White AT, Aziz MA, et al. Comparison of ivermectin and diethylcarbamazine in the treatment of onchocerciasis. N Engl J Med. 1985;313(3):133-8.

8. Lariviere M, Vingtain P, Aziz M, Beauvais B, Weimann D, Derouin F, et al. Double-blind study of ivermectin and diethylcarbamazine in African onchocerciasis patients with ocular involvement. Lancet. 1985;2(8448):174-7.

9. Dadzie KY, Bird AC, Awadzi K, Schulz-Key H, Gilles HM, Aziz MA. Ocular findings in a double-blind study of ivermectin versus diethylcarbamazine versus placebo in the treatment of onchocerciasis. Br J Ophthalmol. 1987;71(2):78-85.

10. Awadzi K, Dadzie KY, Schulz-Key H, Gilles HM, Fulford AJ, Aziz MA. The chemotherapy of onchocerciasis. XI. A double-blind comparative study of ivermectin, diethylcarbamazine and placebo in human onchocerciasis in northern Ghana. Ann Trop Med Parasitol. 1986;80(4):433-42.

11. Diallo S, Aziz MA, Lariviere M, Diallo JS, op-Mar I, N'Dir O, et al. A double-blind comparison of the efficacy and safety of ivermectin and diethylcarbamazine in a placebo controlled study of Senegalese patients with onchocerciasis. Trans R Soc Trop Med Hyg. 1986;80(6):927-34.

12. White AT, Newland HS, Taylor HR, Erttmann KD, Keyvan-Larijani E, Nara A, et al. Controlled trial and dose-finding study of ivermectin for treatment of onchocerciasis. J Infect Dis. 1987;156(3):463-70.

13. Albiez EJ, Newland HS, White AT, Kaiser A, Greene BM, Taylor HR, et al. Chemotherapy of onchocerciasis with high doses of diethylcarbamazine or a single dose of ivermectin: microfilaria levels and side effects. Trop Med Parasitol. 1988;39(1):19-24.

14. Awadzi K, Dadzie KY, Klager S, Gilles HM. The chemotherapy of onchocerciasis. XIII. Studies with ivermectin in onchocerciasis patients in northern Ghana, a region with long lasting vector control. Trop Med Parasitol. 1989;40(3):361-6.

15. Lariviere M, Beauvais B, Aziz M, Garin JF, Abeloos J, Derouin F, et al. Etude en Cote-d'Ivoire (1985-1987) de l'éfficacité et de la tolérance de l'ivermectin (Mectizan) dans l'onchocercose humaine. I Etude comparative en double insu de 220 sujets onchocerquiens traité par dose unique orale de 100, 150 ou 200 mcg/kg. [A study in the Ivory Coast (1985-1987) of the efficacy and tolerance of ivermectin (Mectizan) in human onchocerciasis. I. A comparative double-blind study of 220 patients with onchocerciasis treated with a single oral dose of 100, 150 or 200 mcg/kg]. Bull Soc Pathol Exot Filiales. 1989;82(1):35-47.

16. Newland HS, White AT, Greene BM, D'Anna SA, Keyvan-Larijani E, Aziz MA, et al. Effect of single-dose ivermectin therapy on human Onchocerca volvulus infection with onchocercal ocular involvement. Br J Ophthalmol. 1988;72(8):561-9.

17. Taylor HR, Semba RD, Newland HS, Keyvan-Larijani E, White A, Dukuly Z, et al. Ivermectin treatment of patients with severe ocular onchocerciasis. Am J Trop Med Hyg. 1989;40(5):494-500.

18. Lariviere M, Beauvais B, Aziz M, Garin JF, Peignot C, Abeloos J, et al. Etude en Cote-d'Ivoire (1985-1987) de l'éfficacité et de la tolérance de l'ivermectin (Mectizan) dans l'onchocercose humaine. III Tolérance et efficacité d'une dose unique orale de 150 mcg/kg chez les enfants [A study in the Ivory Coast (1985-1987) of the efficacy and tolerance of ivermectin (Mectizan) in human onchocerciasis. III. The tolerance and efficacy of a single oral dose of 150 mcg/kg in children]. Bull Soc Pathol Exot Filiales. 1989;82(1):58-64.

19. Awadzi K, Hero M, Opoku NO, Addy ET, Buttner DW, Ginger CD. The chemotherapy of onchocerciasis XVIII. Aspects of treatment with suramin. Trop Med Parasitol. 1995;46(1):19-26.

20. Awadzi K, Addy ET, Opoku NO, Plenge-Bonig A, Buttner DW. The chemotherapy of onchocerciasis XX: ivermectin in combination with albendazole. Trop Med Parasitol. 1995;46(4):213-20.

21. Awadzi K, Edwards G, Duke BO, Opoku NO, Attah SK, Addy ET, et al. The co-administration of ivermectin and albendazole--safety, pharmacokinetics and efficacy against Onchocerca volvulus. Ann Trop Med Parasitol. 2003;97(2):165-78.
